# Synthesis of photo- and ionochromic N-acylated 2-(aminomethylene)benzo[*b*]thiophene-3(2*Н*)-ones with a terminal phenanthroline group

**DOI:** 10.3762/bjoc.20.47

**Published:** 2024-03-11

**Authors:** Vladimir P Rybalkin, Sofiya Yu Zmeeva, Lidiya L Popova, Irina V Dubonosova, Olga Yu Karlutova, Oleg P Demidov, Alexander D Dubonosov, Vladimir A Bren

**Affiliations:** 1 Federal Research Centre the Southern Scientific Centre of the Russian Academy of Sciences, Rostov-on-Don 344006, Russian Federation; 2 Institute of Physical and Organic Chemistry, Southern Federal University, Rostov-on-Don 344090, Russian Federation; 3 North Caucasus Federal University, Stavropol 355009, Russian Federation

**Keywords:** fluorescence, molecular switches, N→O acyl rearrangement, naked eye effect, photochromism

## Abstract

A series of novel photo- and ionochromic N-acylated 2-(aminomethylene)benzo[*b*]thiophene-3(2*Н*)-ones with a terminal phenanthroline receptor substituent was synthesized. Upon irradiation in acetonitrile or DMSO with light of 436 nm, they underwent *Z*–*E* isomerization of the C=C bond, followed by very fast N→O migration of the acyl group and the formation of nonemissive O-acylated isomers. These isomers were isolated preparatively and fully characterized by IR, ^1^H, and ^13^C NMR spectroscopy as well as HRMS and XRD methods. The reverse thermal reaction was catalyzed by protonic acids. N-Acylated compounds exclusively with Fe^2+^ formed nonfluorescent complexes with a contrast naked-eye effect: a color change of the solutions from yellow to dark orange. Subsequent selective interaction with AcO^−^ led to the restoration of the initial absorption and emission properties. Thus, the obtained compounds represent dual-mode “on–off–on” switches of optical and fluorescent properties under sequential exposure to light and H^+^ or sequential addition of Fe^2+^ and AcO^−^ ions.

## Introduction

Photochromism is defined as the reversible transformation of a molecular entity between different forms, having different absorption spectra, induced in one or both directions by absorption of electromagnetic radiation [1–4]. Due to the different structures and the different optical and emission properties of these forms, photochromic compounds are used in 3D optical memory devices, photoswitches of different types, molecular logic gates, photopharmacology, bioimaging and chemosensorics [5–11]. For most photochromic compounds, irradiation of the solution or solid results in a deepening of the color (positive photochromism). Less studied are those characterized by photoinduced bleaching (negative or inverse photochromism), such as merocyanine forms of spiropyrans and spirooxazines, azomethine imines, thioindigoid dyes and N→O acylotropic systems [12–15]. Recently, they have been actively used to create next-generation molecular switches, materials with new properties (in particular, color change depending on the intensity of sunlight), photochromic tags for biological research and optical sensors [15–21].

To develop new dual-mode molecular switches capable of efficient modulation of optical and fluorescent properties, both upon irradiation with visible light and upon sequential addition of Fe^2+^ and AcO^−^, we synthesized N-acylated 2-(aminomethylene)benzo[*b*]thiophene-3(2*Н*)-ones with a terminal phenanthroline substituent and studied the spectral-luminescent, photochromic and ionochromic properties. The phenanthroline moiety was incorporated into the molecule due to the known ability to coordinate with metal cations [22–23]. Dual-mode naked-eye molecular switches controlled by both light and changes in the ionic composition of the medium can be used as elements of electronic devices, for optical recording of information as well as in photopharmacology, light-gated catalysis and bioimaging [24–26]. In addition, such systems provide a convenient platform for creating chemo- and biosensors for rapid analysis of the ionic composition of the environment [27–28].

## Results and Discussion

**Scheme 1:**
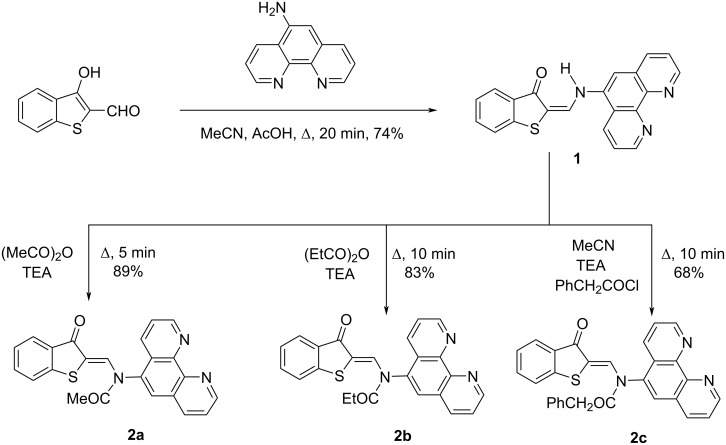
Synthesis of compound **1** and N-acylated compounds **2a**–**c**.

The starting compound for the synthesis of N-acylated 2-(aminomethylene)benzo[*b*]thiophene-3(2*Н*)-ones **2a**–**c** with a terminal phenanthroline substituent was (*E*)-2-(((1,10-phenanthrolin-5-yl)amino)methylene)benzo[*b*]thiophen-3(2*H*)-one (**1**), obtained by condensation of 3-hydroxybenzo[*b*]thiophene-2-carbaldehyde with 5-aminophenanthroline in acetonitrile (Scheme 1). (*Z*)-*N*-((3-Oxobenzo[*b*]thiophen-2(3*H*)-ylidene)methyl)-*N*-(1,10-phenanthrolin-5-yl)acetamide (**2a**) and (*Z*)-*N*-((3-oxobenzo[*b*]thiophen-2(3*H*)-ylidene)methyl)-*N*-(1,10-phenanthrolin-5-yl)propionamide (**2b**) were prepared by short-term boiling of **1** in acetic or propionic anhydride, respectively. To obtain (*Z*)-*N*-((3-oxobenzo[*b*]thiophen-2(3*H*)-ylidene)methyl)-*N*-(1,10-phenanthrolin-5-yl)-2-phenylacetamide (**2c**), a suspension of **1** in acetonitrile was boiled with phenylacetyl chloride until completely dissolved (Scheme 1 and Supporting Information File 1).

The obtained compounds **2a**–**c** existed as an N-acylated keto form. In the IR spectra, stretching vibrations of the thiophene and amide carbonyl groups were observed at 1663–1678 and 1705–1713 cm^−1^, respectively. The ^1^H NMR spectra contained signals of methine protons (=CH–) in the region 7.92–9.02 ppm, which corresponded to the *Z*-configuration of the C=C bond. According to data previously obtained [14], the signals of methine protons of *E*-isomers should be in the region of approximately 5.90 ppm [14]. Other IR, ^1^Н and ^13^С NMR spectroscopy and HRMS data confirming the structure of the synthesized compounds **1** and **2a**–**c** are presented in Supporting Information File 2.

Nonacylated compound **1** showed long-wavelength absorption at 458 nm, while acylation led to a hypsochromic shift of the maximum in compounds **2a**–**c** to 423–426 nm (Table 1). The intensity of this absorption band decreased with increasing steric hindrance in the order R = acetyl (i.e., **2a**) > propionyl (i.e., **2b**) > phenylacetyl (i.e., **2c**).

**Table 1:** Absorption and fluorescence spectra of compounds **1**, **2a** and **2b** in acetonitrile and compound **2c** in DMSO, and quantum yields of the **2a**–**c→3a**–**c** rearrangement^a^.

compound	λ_abs_, nm (ε, L⋅mol^−1^⋅cm^−1^)	λ_fl_, nm (φ_fl_)	φ**_2_**_→_**_3_**

**1**	344 (12800), 458 (20000)	506 (0.12)	—
**2a**	303 (25000), 423 (14000)	468 (0.16)	0.34
**2b**	300 (18800), 426 (9200)	465 (0.15)	0.35
**2c**	309 (24600), 425 (6800)	467 (0.13)	0.40

^a^λ_abs_ and λ_fl_: maxima of the absorption and fluorescence bands, respectively. φ_fl_: quantum yield of fluorescence. φ**_2_**_→_**_3_**: overall quantum yield for stepwise N→O rearrangement. *c* 5.0 × 10^−5^ mol⋅L^−1^. λ_ex_ 420 nm (455 nm for **1**). PMT voltage 800 V.

**Figure 1:**
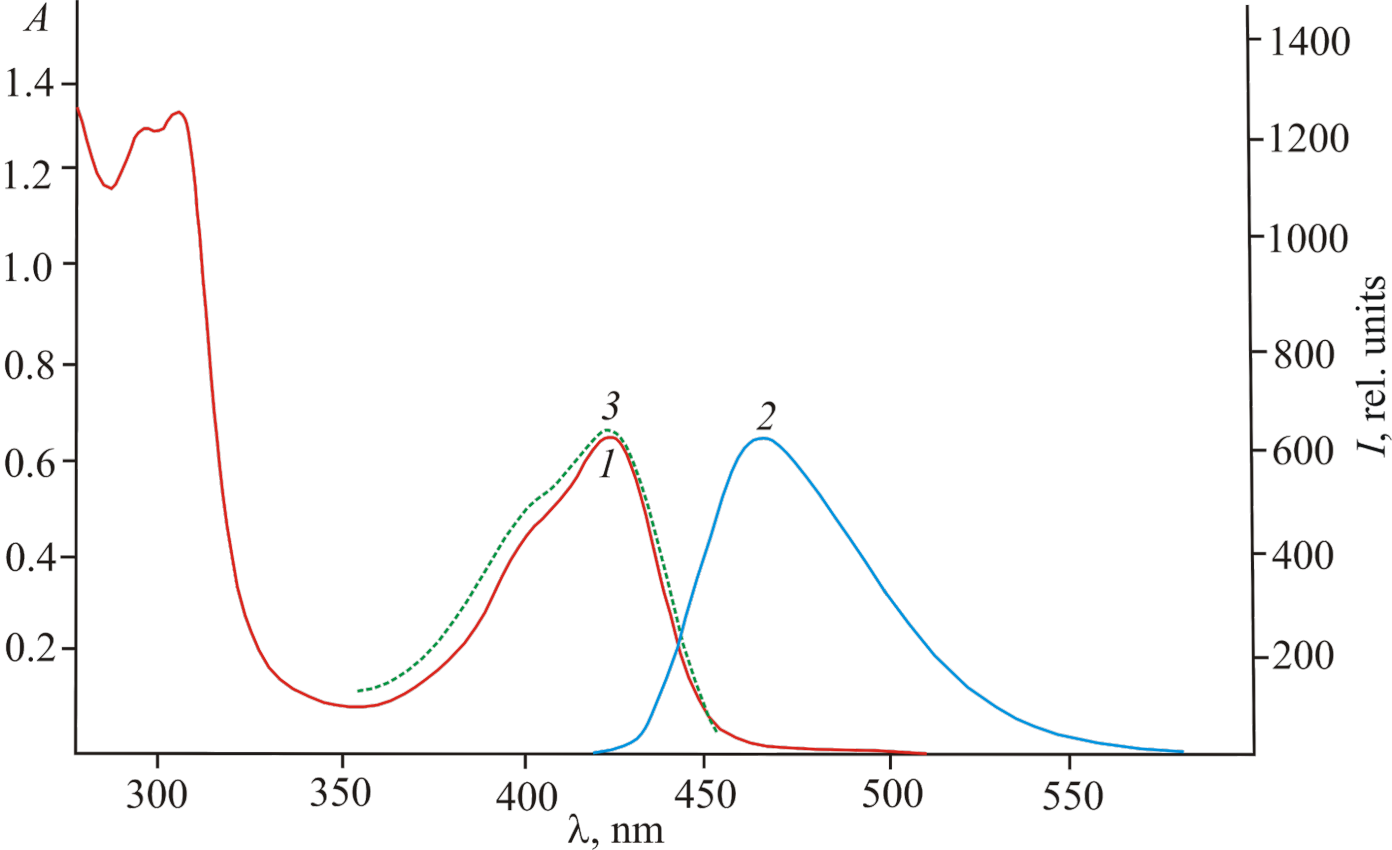
Absorption (1), fluorescence (2, λ_ex_ = 410 nm) and fluorescence excitation (3, λ_fl_ = 465 nm) spectra of compound **2a** in acetonitrile (*c* 5.0 × 10^−5^ mol·L^−1^).

N-Acylated compounds **2a**–**c** in solutions exhibited fluorescence in the region of 465–468 nm, and the excitation emission spectra agreed well with the absorption spectra (Figure 1).

**Figure 2:**
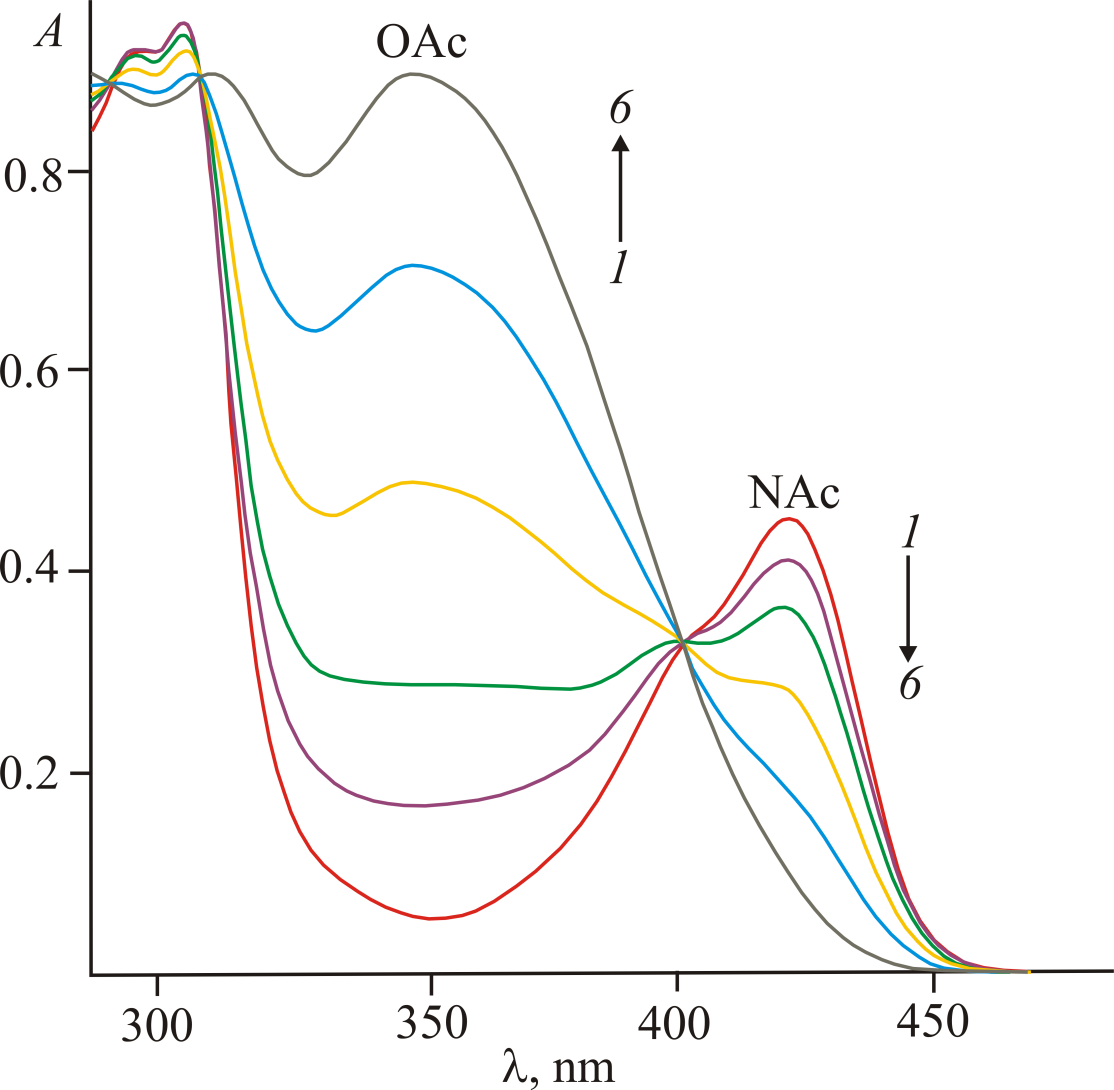
Electronic absorption spectra of compound **2b** in acetonitrile before (1) and after 15 s (2), 35 s (3), 75 s (4), 2.5 min (5) and 5 min (6) of irradiation with light of 436 nm (*c* 5.0 × 10^−5^ mol·L^−1^).

**Scheme 2:**
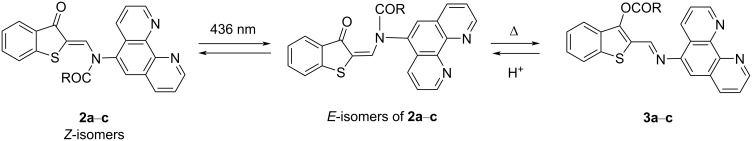
Photoisomerization of N-acylated ketoenamines **2a**–**c**.

Compounds **2a**–**c** in solutions demonstrated a typical negative photochromism [1,16] when irradiated with visible light of 436 nm (Figure 2). A decrease in the long-wavelength absorption was accompanied by a simultaneous increase in the shorter-wavelength absorption in the spectral region around 370 nm. During this process, a gradual diminishment of the initial fluorescence intensity at 465–468 nm up to zero was observed. The resulting O-acylated isomers **3a**–**c** were nonemissive. According to previous findings, the **2a**–**c**→**3a**–**c** transformation is a two-step process: 1) *Z*–*E* photoisomerization and 2) extremely fast nonadiabatic N→O acyl group transfer (Scheme 2) [14,16]. These two stages occur almost simultaneously, and hence the term “overall quantum yield” is used in Table 1 [29]. The formation of the short-lived *E*-isomer was earlier observed only in vitrified solvents at 77 K or under flash photolysis conditions [14]. As such, the parameters of the thermal acyl migration under steady-state irradiation conditions were not determined.

The reverse reaction **3a**–**c→2a**–**c** with full restoration of the initial absorption and fluorescence properties was characterized by a high activation barrier and could be accomplished by heating a solution of **3a**–**c** in *o*-dichlorobenzene at 423 K or by passing dry hydrogen chloride through a solution of **3a**–**c** in acetonitrile. However, the simplest and most effective method was the addition of a catalytic amount of HClO_4_ [14].

Although studies on photoacylotropic systems have been carried out for some time [14,16], only within the framework of this work, a method for the preparative synthesis of photoproducts **3a**–**c** under the influence of irradiation was developed. We applied a modified procedure using a Sweko IP65 LED emitter that had been previously developed in our studies for similar tasks [30]. For this purpose, a suspension of a yellow solid **2a**–**c** in acetonitrile was boiled for 10–15 s and then irradiated with an emitter for 3–5 min. This process was repeated up to 10 times until complete dissolution had occurred. The colorless solids **3a** (80%), **3b** (75%) and **3c** (85%), respectively, gradually precipitated. For the first time in the course of studying N→O acylotropic migrations, photorearrangement products **3a**–**c** were comprehensively characterized by IR, ^1^Н and ^13^C NMR spectroscopy, HRMS (Supporting Information File 2) as well as by X-ray diffraction analysis.

**Figure 3:**
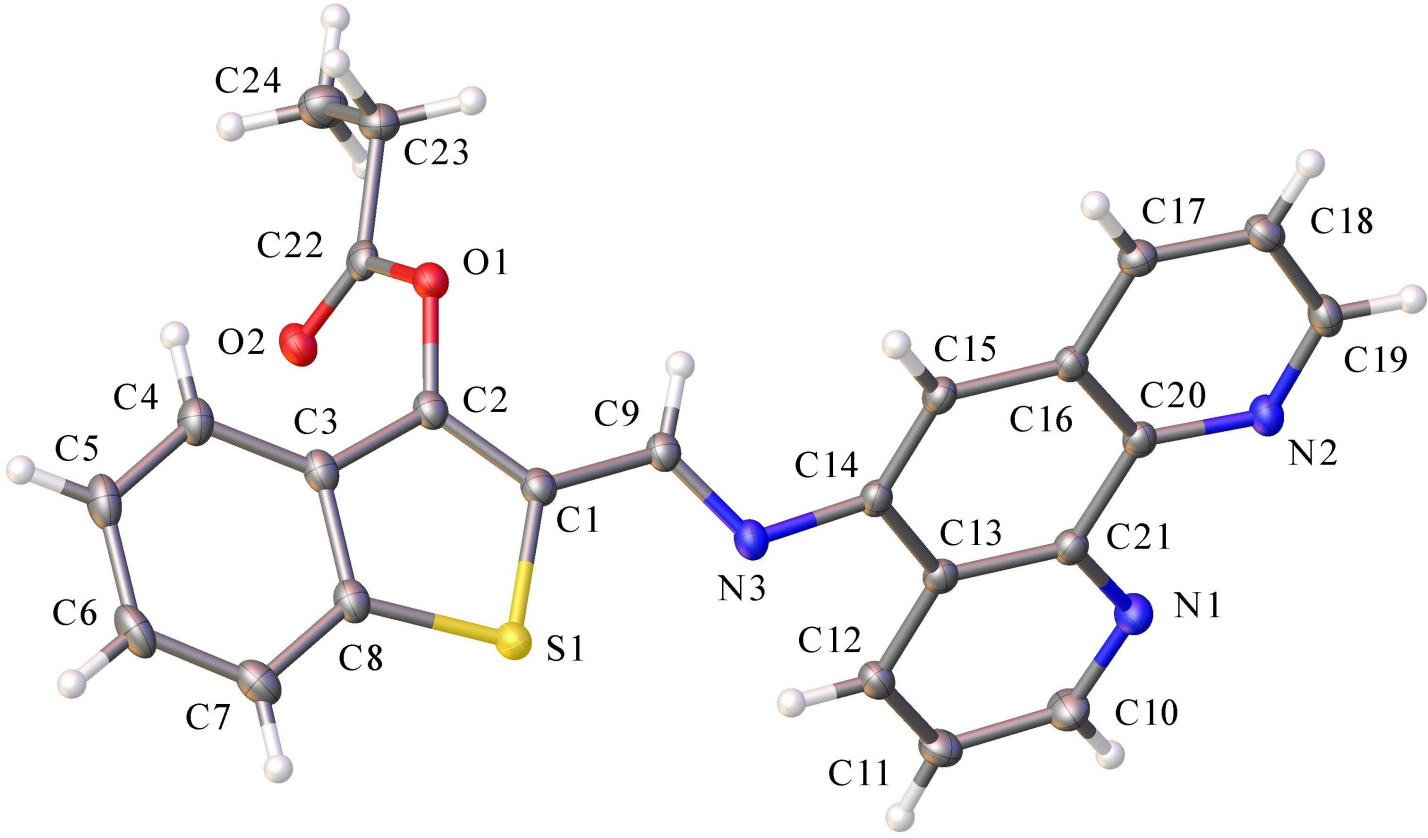
Molecular structure of O-acylated isomer **3b**. Thermal ellipsoids are drawn at the 50% probability level.

The molecular structure of **3b** is shown in Figure 3. The crystal data, details of the data collection and refinements for **3b** as well as complete lists of bond lengths and bond angles are given in Tables S1–S4, Supporting Information File 3.

Compound **3b** had the structure of an O-acylated isomer and possessed an *E*-s-*cis*(S,N) conformation relative to the C(l)–C(9) bond (Figure 3). The benzo[*b*]thiophene fragment was planar, whereas the propionyl group COCH_2_CH_3_ was not coplanar with this plane. This was due to a torsional rotation around the O(l)–C(2) bond by the C(22)–O(1)–C(2)–C(1) fragment with an angle of 105.89°. The phenanthroline moiety was planar and rotated relative to the benzo[*b*]thiophene part of the molecule (plane twist angle 122.73°, fold angle 4.38°).

**Figure 4:**
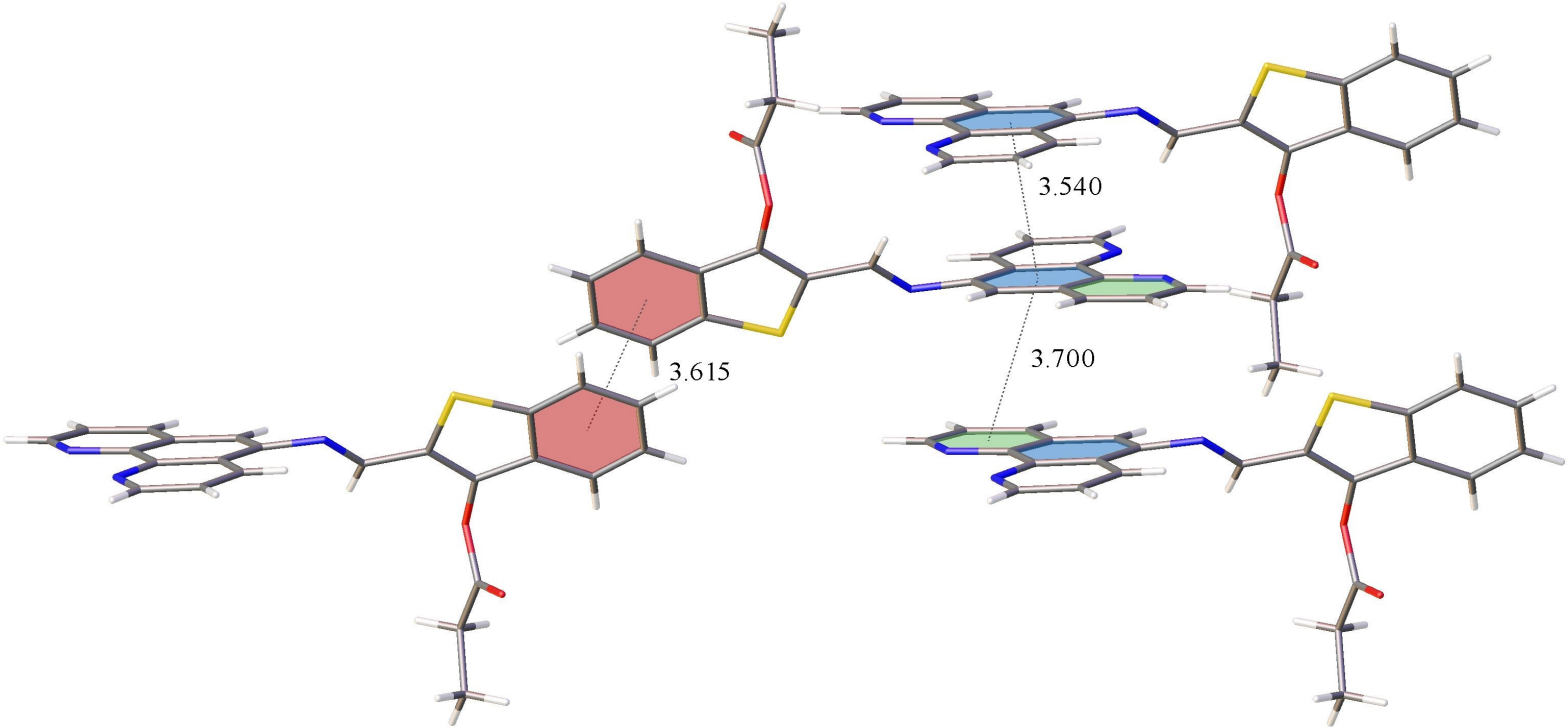
Fragment of the molecular packing of compound **3b**, showing π–π interactions in the crystalline state. The main centroid–centroid distances are indicated in Å.

The molecular packing of compound **3b** was characterized by the presence of numerous π–π interactions (Figure 4).

Intermolecular interactions in the benzo[*b*]thiophene fragment (red planes in Figure 4) were characterized by the following parameters: plane centroid–plane centroid distance 3.6153(10) Å (shift 1.6063(18) Å, twist and fold angles 0.0°). The closest contact in the phenanthroline fragment (blue plane–blue plane, top fragment in Figure 4) had a plane centroid–plane centroid distance of 3.5398(9) Å (shift 0.4273(18) Å, twist and fold angles 0.00°). One pyridine cycle of the phenanthroline unit was also involved in a π–π-stacking interaction (blue plane–green plane in Figure 4), with the plane centroid–plane centroid distance being 3.6998(8) Å (plane shift 1.4919(17) Å, twist and fold angles 1.54° and 1.92°, respectively).

**Figure 5:**
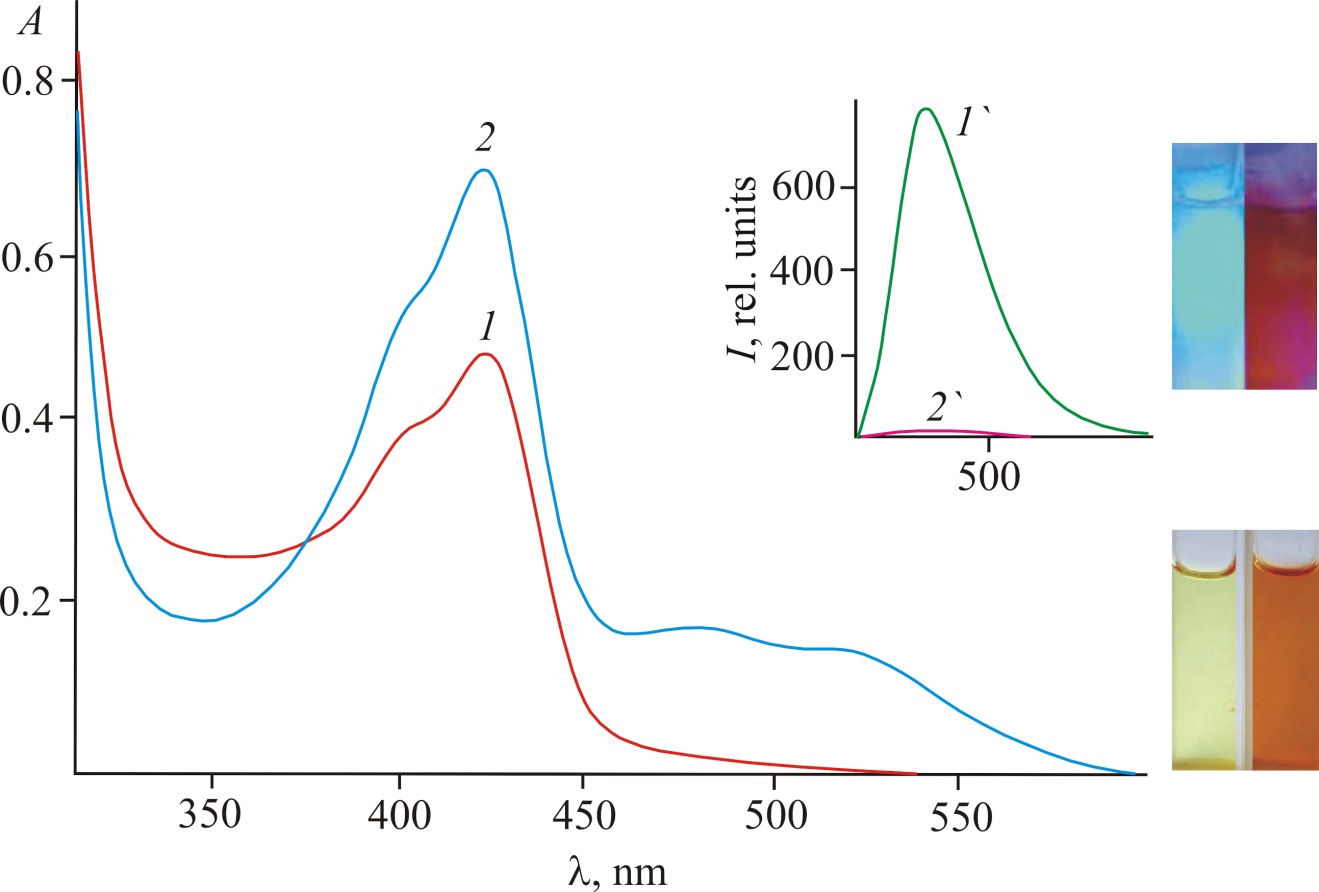
Absorption spectra of compound **2a** in acetonitrile before (1) and after (2) the addition of Fe^2+^ (*c***_2a_** 5.0 × 10^−5^ mol·L^−1^, *c*_Fe2+_ 1.0 × 10^−4^ mol·L^−1^). Inset: fluorescence spectra before (1’) and after (2’) the addition of Fe^2+^ (λ_ex_ = 422 nm). The photographs show absorption (bottom right) and fluorescence (upon irradiation with light of 436 nm, top right) before and after the addition of Fe^2+^.

**Figure 6:**
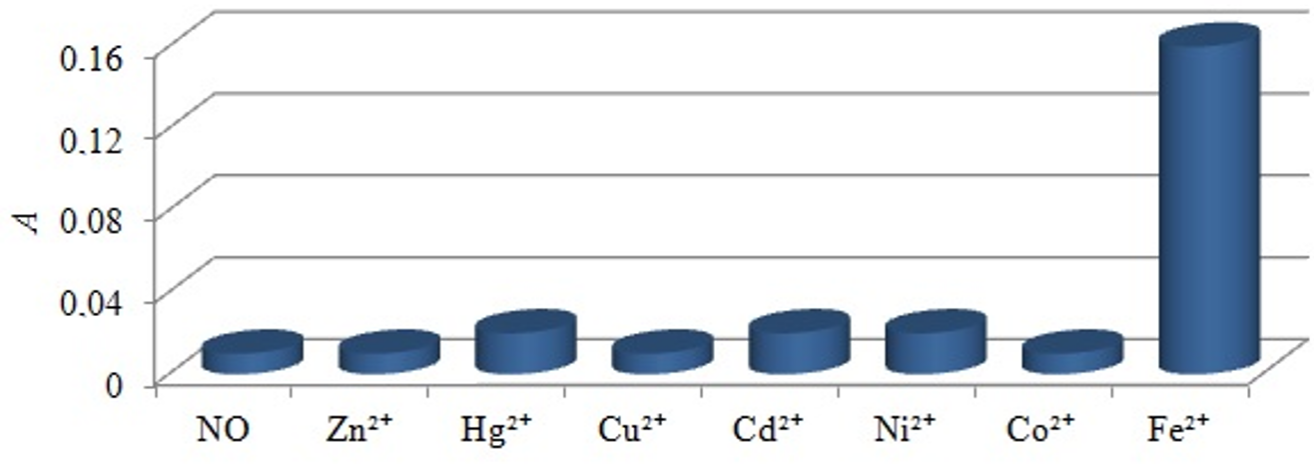
Changes in the absorption intensity of compound **2a** in acetonitrile at 520 nm after the addition of metal perchlorates (*с***_2a_** 5 × 10^−5^ mol·L^−1^, *с*_M2+_ 1.0 × 10^−4^ mol·L^−1^).

Cation-induced transformations of the absorption and fluorescence spectra of **2a**–**c** were studied by the action of d-metal perchlorates (Zn^2+^, Hg^2+^, Cu^2+^, Cd^2+^, Ni^2+^, Co^2+^ and Fe^2+^) in acetonitrile (**2a** and **2b**) and DMSO (**2c**). Exclusively Fe^2+^ caused an appearance of new broad long-wavelength absorption bands at 480–530 nm with a contrast naked-eye effect: a visually distinguishable color change of the solutions from yellow to dark orange (Figure 5). Other cations did not demonstrate a measurable effect (Figure 6). Complexes **2a**–**c** with Fe^2+^ in acetonitrile and DMSO were nonfluorescent.

**Scheme 3:**
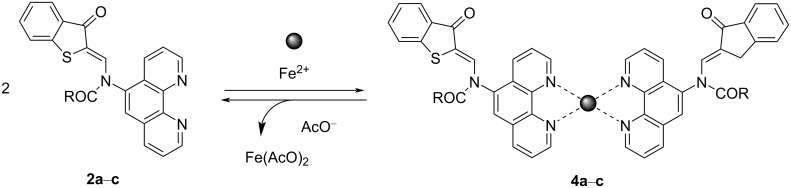
Sequential interaction of compounds **2a**–**c** with Fe^2+^ and AcO^−^.

**Figure 7:**
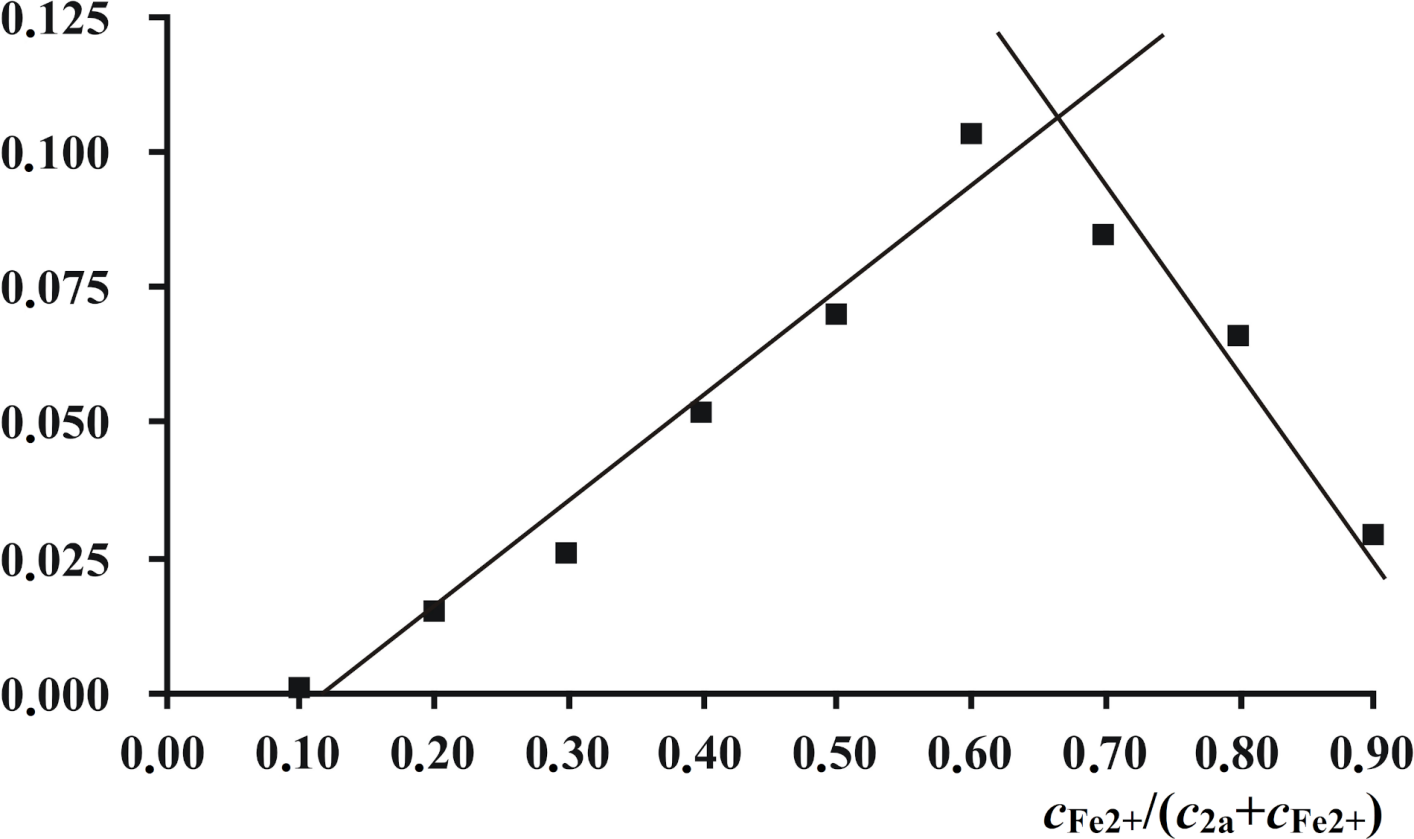
Job’s plot at the wavelength 429 nm, reflecting the interaction of compound **2a** with Fe^2+^ in acetonitrile. The total concentration *c***_2a_** + *c*_Fe2+_ was 1.5 × 10^−4^ mol·L^−1^.

According to spectrophotometric titration data and the isomolar series method, compounds **2a**–**c** formed the 2:1 complexes **4a**–**c** with Fe^2+^ (Scheme 3 and Figure 7).

**Figure 8:**
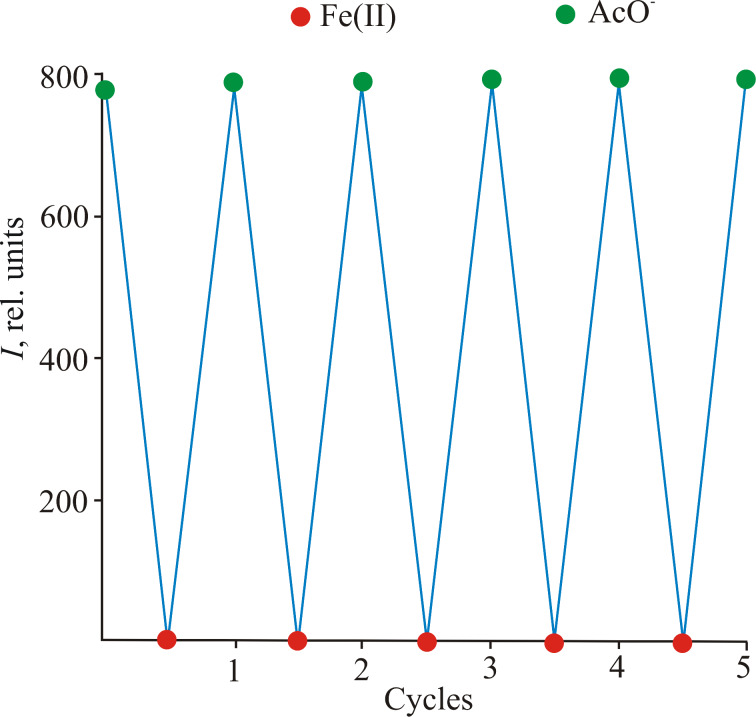
Fluorescence intensity of compound **2a** upon alternate addition of Fe^2+^ and AcO^−^.

It was found that selective interaction of the resulting in situ complex **4a** with AcO^−^ led to restoration of the initial absorption and emission properties [31–32]. Other tetra-*n*-butylammonium salts (TBAX, X = F, Cl, Br, I, CN, SCN, NO_3_) did not cause similar changes in the absorption spectra. This process could be carried out at least 4 or 5 times, which allows modulating optical and fluorescent properties by sequentially adding Fe^2+^ and AcO^−^ to the acetonitrile solution of compound **2a** (Figure 8).

## Conclusion

A series of novel photo- and ionochromic N-acylated 2-(aminomethylene)benzo[*b*]thiophene-3(2*Н*)-ones with a terminal phenanthroline receptor substituent was synthesized. Upon irradiation with light of 436 nm, the resulting compounds in solutions exhibited negative photochromism due to *Z*–*E* photoisomerization of the C=C bond, followed by very fast thermal N→O migration of the acyl group and the formation of O-acylated isomers. This rearrangement was accompanied by a decrease of the initial fluorescence intensity at 465–468 nm up to zero, since the resulting OAc^−^ form was nonemissive. The reverse reaction occurred catalytically in the presence of HClO_4_.

A special technique for the preparative synthesis of photoproducts was developed. For the first time in the course of studying N→O acylotropic migrations, O-acylated photoproducts were comprehensively characterized by IR, ^1^H and ^13^C NMR spectroscopy, HRMS and X-ray diffraction analysis. Selectively, Fe^2+^ caused an appearance of new broad long-wavelength absorption bands at 480−530 nm with a contrast naked-eye effect: a visually distinguishable color change of the solutions from yellow to dark orange. The obtained complexes with Fe^2+^ in acetonitrile and DMSO were nonfluorescent. They selectively interacted with AcO^−^, which led to the restoration of the initial absorption and emission properties. Thus, the obtained compounds were dual-mode “on−off−on” switches of the fluorescent properties upon sequential exposure to light and H^+^ as well as sequential addition of Fe^2+^ and AcO^−^.

## Experimental

### General

The ^1^H and ^13^C NMR spectra were recorded on an integrated analytical LC–SPE–NMR–MS system AVANCE-600 (Bruker, 600 MHz for ^1^H and 150.96 MHz for ^13^C) in CDCl_3_. The signals were referred to with respect to the signals of residual protons of deuterated solvent (7.24 ppm). IR spectra were recorded on an FT/IR-6800 FTIR spectrometer (JASCO). The IR and NMR spectra were recorded using equipment from the Shared Use Centre “Molecular spectroscopy” of the Southern Federal University. Electronic absorption spectra were obtained on a Varian Cary 100 spectrophotometer. Electronic emission spectra were recorded on a Varian Cary Eclipse spectrofluorimeter. Acetonitrile and DMSO (spectral pure grade), Cd^2+^, Hg^2+^, Cu^2+^, Zn^2+^, Ni^2+^, Co^2+^ and Fe^2+^ perchlorates as well as TBAX (X = F, Cl, Br, I, CN, SCN, NO_3_) salts (Aldrich) were used to prepare the solutions. The solutions (in a quartz cell, *l* = 1 cm) were irradiated with filtered light from a high-pressure Hg lamp on a Newport 66941 equipment supplied with a set of interference light filters. The light intensity was 6.4 × 10^16^ photons⋅s^−1^ for the 436 nm spectral line. (*Z*)-*N*-((3-Oxobenzo[*b*]thiophen-2(3*H*)-ylidene)methyl)-*N*-phenylacetamide was used as an actinometer for the quantum yield calculations (φ_fl_ = 0.60 ± 0.005) [14,33]. For preparative purposes, a Sweko IP65 led emitter (SUL-S1-20W-230-4000K-WH) was used. Spectral-fluorescent experiments were performed using solutions in acetonitrile or DMSO in quartz cells (*l* = 1 cm, *V* = 2 mL). Fluorescence quantum yields were determined relatively to (*Z*)-*N*-((3-oxobenzo[*b*]thiophen-2(3*H*)-ylidene)methyl)-*N*-phenylacetamide as a standard (φ_fl_ = 0.16 ± 0.005) [14,33]. Stock solutions of compounds **2a**–**c** (*c* 1.0 × 10^–4^ mol⋅L^−1^) and metal perchlorates (*c* 2.0 × 10^–4^ mol⋅L^−1^) were used. 1 mL of a **2a**–**c** solution and 1 mL of a perchlorate solution were mixed directly in the cuvette and thoroughly stirred. Hence, the working concentration of the compounds **2**–**c** and the cations was 5.0 × 10^–5^ mol⋅L^−1^ and 1.0 × 10^−5^ mol⋅L^−1^. HRMS analysis was performed on a Bruker UHR-TOF Maxis™ Impact instrument (electrospray ionization). Melting points were determined on a Fisher–Johns melting point apparatus.

### X-ray diffraction study

The X-ray diffraction dataset of compound **3b** was recorded on an Agilent SuperNova diffractometer using a microfocus X-ray radiation source with copper anode and Atlas S2 two-dimensional CCD detector. Crystal data for C_24_H_17_N_3_O_2_S (*M* = 411.46 g⋅mol^−1^): triclinic, space group *P−*1 (no. 2), *a* = 7.43030(10) Å, *b* = 9.6398(2) Å, *c* = 14.3294(3) Å, α = 75.731(2)°, β = 82.686(2)°, γ = 78.664(2)°, *V* = 971.93(3) Å^3^, *Z* = 2, *T* = 293(2) K, μ (Cu Kα) = 1.701 mm^−1^, *D*_calc_ = 1.406 g/cm^3^, 17777 reflections measured (9.61° ≤ 2Θ ≤ 152.768°), 4053 unique (*R*_int_ = 0.0201, *R*_σ_ = 0.0153) that were used in all calculations. The final *R*_1_ was 0.0307 (*I* ≥ 2σ(*I*)) and *wR*_2_ was 0.0813 (all data).

Reflections were recorded and unit cell parameters were determined and refined using the dedicated CrysAlisPro software suite [34]. The structure was solved with the ShelXT program [35] and refined with the ShelXL program [36], and the graphics were rendered using the Olex2 software suite [37]. The complete X-ray structural dataset for compound **2a** was deposited with the Cambridge Crystallographic Data Centre (CCDC 2299603). The data can be obtained free of charge via https://www.ccdc.cam.ac.uk/structures/.

## Supporting Information

File 1 Experimental procedures and characterization data for all new compounds **1**, **2a**–**c** and **3a**–**c**.

File 2 ^1^H and ^13^C NMR, IR and HRMS spectra of all novel compounds.

File 3 X-ray analysis data of **3b**.

## Data Availability

All data that supports the findings of this study is available in the published article and/or the supporting information to this article.
