# Supplementary material for: Synthesis of photo- and ionochromic N-acylated 2-(aminomethylene)benzo[b]thiophene-3(2Н)-ones with a terminal phenanthroline group
Source: Beilstein J Org Chem. 2024 Mar 11;20:552–60. doi: 10.3762/bjoc.20.47 (PMC10949002; doi:10.3762/bjoc.20.47)
Supplement: File 1 — Experimental procedures and characterization data for all new compounds 1, 2a–c and 3a–c. [file Beilstein_J_Org_Chem-20-552-s001.pdf]

BEILSTEIN JOURNAL OF ORGANIC CHEMISTRY

Supporting Information

for

Synthesis of photo- and ionochromic N-acylated 2-(aminomethylene)benzo[*b*]thiophene-3(2*H*)-ones with a terminal phenanthroline group

Vladimir P. Rybalkin, Sofiya Yu. Zmeeva, Lidiya L. Popova, Irina V. Dubonosova, Olga Yu. Karlutova, Oleg P. Demidov, Alexander D. Dubonosov and Vladimir A. Bren

Beilstein J. Org. Chem. **2024,** *20,* 552–560. doi:10.3762/bjoc.20.47

Experimental procedures and characterization data for all new compounds 1, 2a–c and 3a–c

License and Terms: This is a supporting information file under the terms of the Creative Commons Attribution License (https://creativecommons.org/licenses/by/4.0). Please note that the reuse, redistribution and reproduction in particular requires that the author(s) and source are credited and that individual graphics may be subject to special legal provisions.
The license is subject to the *Beilstein Journal of Organic Chemistry* terms and conditions: (https://www.beilstein-journals.org/bjoc/terms)

Contents

1. Synthesis S2

2. References S6

Synthesis

(*E*)-2-(((1,10-Phenanthrolin-5-yl)amino)methylene)benzo[*b*]thiophen-3(2*H*)-one (**1**)

5-Aminophenanthroline (1.95 g, 10 mmol) was dissolved in 50 mL of acetonitrile by means of stirring under reflux conditions. To the resulting hot solution, 1.78 g (10 mmol) of 3-hydroxybenzo[*b*]thiophene-2-carbaldehyde [1] was added, followed by 3 mL of acetic acid. The obtained mixture was refluxed for 15–20 min and then cooled to room temperature. The precipitate that formed was filtered, washed with acetonitrile (3 × 5 mL) and recrystallized from DMF. Yield 2.63 g (74%), mp 231–234°C (DMF). IR spectrum, ν, cm^{-1}: 1619 (C=O), 1637 (C=O), 3688-3060 (NH). ^1H NMR spectrum ($CDCl_3$), δ, ppm: 7.31 td ($1H_{Ar}$, J_1 7.4 Hz, J_2 1.0 Hz), 7.40-7.59 m ($2H_{Ar}$, $2H_{phen}$), 7.75 dd ($1H_{phen}$, J_1 8.3 Hz, J_2 4.2 Hz), 7.98 d ($1H_{Ar}$, J 7.6 Hz), 8.10 s (1H, CH), 8.12 dd ($1H_{phen}$, J_1 8.2 Hz, J_2 1.6 Hz), 8.68 dd ($1H_{phen}$, J_1 8.3 Hz, J_2 1.6 Hz), 9.07dd ($1H_{phen}$, J_1 4.3 Hz, J_2 1.7 Hz), 9.24 dd ($1H_{phen}$, J_1 4.3 Hz, J_2 1.5 Hz), 13.31 br. s (1H, NH). ^{13}C NMR spectrum ($CDCl_3$), $δ_C$, ppm: 108.4, 108.7, 122.0, 123.4, 123.7, 123.9, 124.6, 125.6, 128.5, 129.6, 132.9, 133.1, 134.0, 1354.0, 140.2, 144.2, 145.6, 146.7, 149.4, 151.0, 187.1. HRMS (ESI), *m/z*: found, 378.0677 $[M+Na]^+$. $C_{21}H_{13}N_3OS$. Calculated, 378.0964 $[M+Na]^+$.

(*Z*)-*N*-((3-Oxobenzo[*b*]thiophen-2(3*H*)-ylidene)methyl)-*N*-(1,10-phenanthrolin-5-yl)acetamide (**2a**)

Compound **1** (0.1 g, 2.5 mmol) was dissolved in 0.5 mL $(MeCO)_2O$ containing 0.1 mL of triethylamine by means of stirring under reflux conditions. The reaction mixture was refluxed for 5–10 min and then cooled to room temperature. The yellow solid that precipitated after cooling to room temperature was filtered, washed with methanol (3 × 1 mL) and recrystallized from acetic anhydride. Yield 0.1 g (89%), mp 302–304°C ($(MeCO)_2O$). IR spectrum, ν, cm^{-1}: 1663 (C=O), 1705 (C=O). ^1H NMR spectrum ($CDCl_3$), δ, ppm: 2.07 br. s (3H, CH_3), 6.84 d ($1H_{Ar}$, J 8.0 Hz), 7.02 т ($1H_{Ar}$, J 7.5 Hz), 7.27 t ($1H_{Ar}$,

J 6.6 Hz), 7.68 dd (1H_{phen}, J_1 8.3 Hz, J_2 4.3 Hz), 7.74-7.78 m (2H_{phen}), 7.93 s (1H, CH), 8.08 d (1H_{Ar}, *J* 8.2 Hz), 8.35 dd (1H_{phen}, J_1 8.0 Hz, J_2 1.5 Hz), 9.11 br. s (1H_{phen}), 9.27 dd (1H_{phen}, J_1 4.2 Hz, J_2 1.6 Hz), 9.34 dd (1H_{phen}, J_1 4.3 Hz, J_2 1.7 Hz). ^{13}C NMR spectrum ($CDCl_3$), δ_C, ppm: 22.6, 114.2, 123.2, 124.0, 124.3, 125.1, 126.3, 126.6, 127.4, 128.9, 130.5, 130.7, 132.1, 132.4, 134.6, 136.9, 145.7, 146.8, 146.9, 151.6, 152.3, 170.2, 188.8. HRMS (ESI), *m/z*: found, 398.0963 [*M*+H]$^+$. $C_{23}H_{16}N_3O_2S$. Calculated, 398.0958 [*M*+H]$^+$.

(*Z*)-*N*-((3-Oxobenzo[*b*]thiophen-2(3*H*)-ylidene)methyl)-*N*-(1,10-phenanthrolin-5-yl)propionamide (**2b**)

Compound **1** (0.11 g, 2.8 mmol) was dissolved in 0.5 mL $(MeCH_2CO)_2O$ containing 0.1 mL of triethylamine by means of stirring under reflux conditions. The reaction mixture was refluxed for 10 min and then cooled to room temperature. The yellow solid that precipitated after cooling was filtered, washed with acetonitrile (3 × 5 mL) and recrystallized from propionic anhydride. Yield 0.105 g (83%), mp 309–310°C ($(C_2H_5CO)_2O$. IR spectrum, ν, cm^{-1}: 1665 (C=O), 1712 (C=O). ^{1}H NMR spectrum ($CDCl_3$), δ, ppm: 1.09 t (3H, CH_3), 2.12 br. s (1H, $C\underline{H}_2$), 2.45 br. s (1H, $C\underline{H}_2$), 6.84 d (1H_{Ar}, *J* 8.0 Hz), 7.08 t (1H_{Ar}, *J* 8.4 Hz), 7.26 t (1H, Ar, *J* 6.5 Hz),7.68 dd (1H, Het, J_1 8.3 Hz, J_2 4.3 Hz), 7.75-7.78 m (2H_{phen}), 7.92 s (1H, CH), 8.06 dd (1H_{phen}, J_1 8.3 Hz, J_2 1.6 Hz), 8.35 dd (1H_{phen}, J_1 8.0 Hz, J_2 1.7 Hz), 9.15 br. s (1H_{phen}), 9.27 dd (1H_{phen}, J_1 4.3 Hz, J_2 1.6 Hz), 9.34 dd (1H_{phen}, J_1 4.3 Hz, J_2 1.6 Hz). ^{13}C NMR spectrum ($CDCl_3$), δ_C, ppm: 8.9, 28.2, 113.9, 123.2, 124.0, 124.3 125.0, 126.3, 126.8, 127.4, 129.0, 130.6, 130.7, 132.1, 132.4, 134.45, 136.9, 145.8, 146.8, 146.9, 151.6, 152.3, 173.7, 188.8. HRMS (ESI), *m/z*: found, 412.1121 [*M*+H]$^+$. $C_{24}H_{18}N_3O_2S$. Calculated, 412.1114 [*M*+H]$^+$.

(*Z*)-*N*-((3-Oxobenzo[*b*]thiophen-2(3*H*)-ylidene)methyl)-*N*-(1,10-phenanthrolin-5-yl)-2-phenylacetamide (**2c**)

To a suspension of compound **1** (0.6 g, 1.68 mmol) in 200 mL of acetonitrile containing 1.5 mL of triethylamine, 0.75 mL of phenylacetyl chloride was added at room temperature while stirring. The reaction mixture was refluxed for 10–15 min until the precipitate dissolved and then cooled to room temperature. The solution was left at room temperature for 10 h. Gradually, a fine crystalline yellow solid formed. The precipitate was filtered, washed with acetonitrile (3 × 1 mL) and recrystallized from DMF. Yield 0.54 g (68%), mp 301–302°C (DMF). IR spectrum, ν, cm^{-1}: 1668 (C=O), 1731 (C=O). ^1H NMR spectrum ($CDCl_3$), δ, ppm: 3.40-3.80 br. m (2H, CH_2), 6.70-6.90 br. m ($3H_{Ar}$), 7.05-7.18 m ($4H_{Ar}$), 7.26 m ($1H_{Ar}$), 7.54 dd ($1H_{phen}$, J_1 8.3 Hz, J_2 4.3 Hz), 7.68 s ($1H_{phen}$), 7.74-7.76 m ($1H_{Ar}$, $1H_{phen}$), 7.90 br m ($1H_{Ar}$), 8.22 dd ($1H_{phen}$, J_1 8.0 Hz, J_2 1.6 Hz), 9.15 br. s (1H, CH), 9.22 dd ($1H_{phen}$, J_1 4.3 Hz, J_2 1.6 Hz), 9.35 dd ($1H_{phen}$, J_1 4.3 Hz, J_2 1.7 Hz). ^{13}C NMR spectrum ($CDCl_3$), $δ_C$, ppm: 41.9, 114.5, 123.1, 124.0, 124.1, 125.0, 126.3, 126.7, 127.1, 127.4, 128.6, 128.7, 129.6, 130.4, 130.6, 131.6, 132.3, 132.9, 134.5, 136.88, 145.7, 146.6, 146.7, 151.4, 152.3, 170.7, 188.7. HRMS (ESI), *m/z*: found, 474.1272 $[M+H]^+$. $C_{29}H_{20}N_3O_2S$. Calculated, 474.1271 $[M+H]^+$.

O-Acylated photoproducts **3a**–**c**

A suspension of a yellow solid **2a**–**c** (20 mg) in 1 mL of acetonitrile was refluxed for 10–15 s and then irradiated with a Sweko IP65 led emitter (SUL-S1-20W-230-4000K-WH) for 3–5 min. This procedure was repeated up to 10 times until the starting material was completely dissolved. The colorless solid **3a**–**c** gradually precipitated. Then the solid was filtered and washed with acetonitrile (3 × 0.5 mL).

(E)-2(((1,10-Phenanthrolin-5-yl)imino)methyl)benzo[*b*]thiophen-3-yl acetate (**3a**)

Yield 16 mg (80%), mp 219–221°C (CH_3CN). IR spectrum, ν, cm^{-1}: 1757 (C=O). ^1H NMR spectrum ($CDCl_3$), δ, ppm: 2.47 s (3H, OMe), 7.31 s ($1H_{phen}$) 7.41 t ($1H_{Ar}$, J 7.6 Hz), 7.47 t ($1H_{Ar}$, J 7.4 Hz), 7.56-7.61 m ($2H_{phen}$), 7.65 dd ($1H_{phen}$, J_1 8.2 Hz, J_2 4.2 Hz), 7.83 d ($1H_{Ar}$, J 8.0 Hz), 8.20 dd ($1H_{phen}$, J_1 8.0 Hz, J_2 1.2 Hz), 8.73 dd ($1H_{phen}$, J_1 8.2 Hz, J_2 1.4 Hz), 8.77 s (1H, CH), 9.10 dd ($1H_{phen}$, J_1 4.1 Hz, J_2 1.3 Hz) 9.20 dd ($1H_{phen}$, J_1 4.1 Hz, J_2 1.4 Hz). ^{13}C NMR spectrum ($CDCl_3$), δ_C, ppm: 20.6, 110.9, 121.7, 123.0, 123.3, 123.4, 125.0, 126.0, 127.8, 128.7, 128.9, 132.6, 132.8, 135.8, 138.7, 144.5, 145.5, 146.3, 147.1, 149.5, 150.79, 151.4, 168.3. HRMS (ESI), *m/z*: found, 398.0962 $[M+H]^+$. $C_{23}H_{16}N_3O_2S$. Calculated, 398.0958 $[M+H]^+$.

(E)-2(((1,10-Phenanthrolin-5-yl)imino)methyl)benzo[*b*]thiophen-3-yl propionate (**3b**)

Yield 15 mg (75%), mp 225–227°C (CH_3CN). IR spectrum, ν, cm^{-1}: 1764 (C=O). ^1H NMR spectrum ($CDCl_3$), δ, ppm: 1.36 t (3H, Me, J 7.6 Hz), 2.79 q (2H, OCH_2, J 7.6 Hz), 7.31 s ($1H_{phen}$), 7.41 t ($1H_{Ar}$, J 7.6 Hz), 7.47 t ($1H_{Ar}$, J 7.7 Hz), 7.57-7.60 m ($2H_{phen}$), 7.65 dd ($1H_{phen}$, J_1 8.2 Hz, J_2 4.3 Hz), 7.83 d ($1H_{Ar}$, J 8.1 Hz), 8.22 dd ($1H_{phen}$, J_1 8.0 Hz, J_2 1.6 Hz), 8.74 dd ($1H_{phen}$, J_1 8.2 Hz, J_2 1.7 Hz), 8.76 s (1H, CH), 9.11 dd ($1H_{phen}$, J_1 4.3 Hz, J_2 1.7 Hz) 9.21dd ($1H_{phen}$, J_1 4.3 Hz, J_2 1.7 Hz). ^{13}C NMR spectrum ($CDCl_3$), δ_C, ppm: 9.3, 27.5, 110.9, 121.7, 123.0, 123.2, 123.4, 125.0, 126.0, 127.7, 128.8, 128.8, 132.7, 132.8, 135.8, 138.7, 144.7, 145.5, 146.4, 147.2, 149.5, 150.8, 151.4, 171.9. HRMS (ESI), *m/z*: found, 412.1119 $[M+H]^+$. $C_{24}H_{18}N_3O_2S$. Calculated, 412.1114 $[M+H]^+$.

(E)-2(((1,10-Phenanthrolin-5-yl)imino)methyl)benzo[*b*]thiophen-3-yl phenylacetate (**3c**)

Yield 17 mg (85%), mp 221–224°C (CH_3CN). IR spectrum, ν, cm^{-1}: 1750 (C=O). ^1H NMR spectrum ($CDCl_3$), δ, ppm: 4.00 s (2H, OCH_2), 7.09 s ($1H_{phen}$), 7.16-7.46 м ($8H_{Ar}$), 7.60-7.66 m ($2H_{Ar}$), 7.80 d ($1H_{Ar}$, J 8.1 Hz), 8.19 dd ($1H_{phen}$, J_1 8.0 Hz, J_2 1.5 Hz), 8.45 s (1H,

CH), 8.71 dd ($1H_{phen}$, J_1 8.2 Hz, J_2 1.7 Hz), 9.13 dd ($1H_{phen}$, J_1 4.3 Hz, J_2 1.6 Hz), 9.20 dd ($1H_{phen}$, J_1 4.2 Hz, J_2 1.4 Hz). ^{13}C NMR spectrum ($CDCl_3$), δ_C, ppm: 41.4, 110.9, 121.6, 123.0, 123.2, 123.3, 125.1, 126.1, 127.7, 127.8, 128.7, 128.9, 129.0, 129.3, 132.6, 132.8, 133.0, 135.7, 138.7, 144.6, 145.5, 146.3, 146.9, 149.5, 150.8, 151.2, 169.0. HRMS (ESI), *m/z*: found, 474.1273 $[M+H]^+$. $C_{29}H_{20}N_3O_2S$. Calculated, 474.1271 $[M+H]^+$.

References

[1] Bren, V. A.; Usacheva, V. I.; Minkin, V. I. *Chem. Heterocycl. Compd.* **1972,** *8,* 836–840. doi:10.1007/BF00475214
