# Supplementary material for: Synthesis of photo- and ionochromic N-acylated 2-(aminomethylene)benzo[b]thiophene-3(2Н)-ones with a terminal phenanthroline group
Source: Beilstein J Org Chem. 2024 Mar 11;20:552–60. doi: 10.3762/bjoc.20.47 (PMC10949002; doi:10.3762/bjoc.20.47)
Supplement: File 2 — 1H and 13C NMR, IR and HRMS spectra of all novel compounds. [file Beilstein_J_Org_Chem-20-552-s002.pdf]

BEILSTEIN JOURNAL OF ORGANIC CHEMISTRY

Supporting Information

for

Synthesis of photo- and ionochromic N-acylated 2-(aminomethylene)benzo[*b*]thiophene-3(2*H*)-ones with a terminal phenanthroline group

Vladimir P. Rybalkin, Sofiya Yu. Zmeeva, Lidiya L. Popova, Irina V. Dubonosova, Olga Yu. Karlutova, Oleg P. Demidov, Alexander D. Dubonosov and Vladimir A. Bren

Beilstein J. Org. Chem. **2024,** *20,* 552–560. doi:10.3762/bjoc.20.47

^{1}H and ^{13}C NMR, IR and HRMS spectra of all novel compounds

License and Terms: This is a supporting information file under the terms of the Creative Commons Attribution License (https://creativecommons.org/licenses/by/4.0). Please note that the reuse, redistribution and reproduction in particular requires that the author(s) and source are credited and that individual graphics may be subject to special legal provisions.
The license is subject to the *Beilstein Journal of Organic Chemistry* terms and conditions: (https://www.beilstein-journals.org/bjoc/terms)

Figure S1: ^{1}H NMR spectrum of compound **1** in $CDCl_3$.

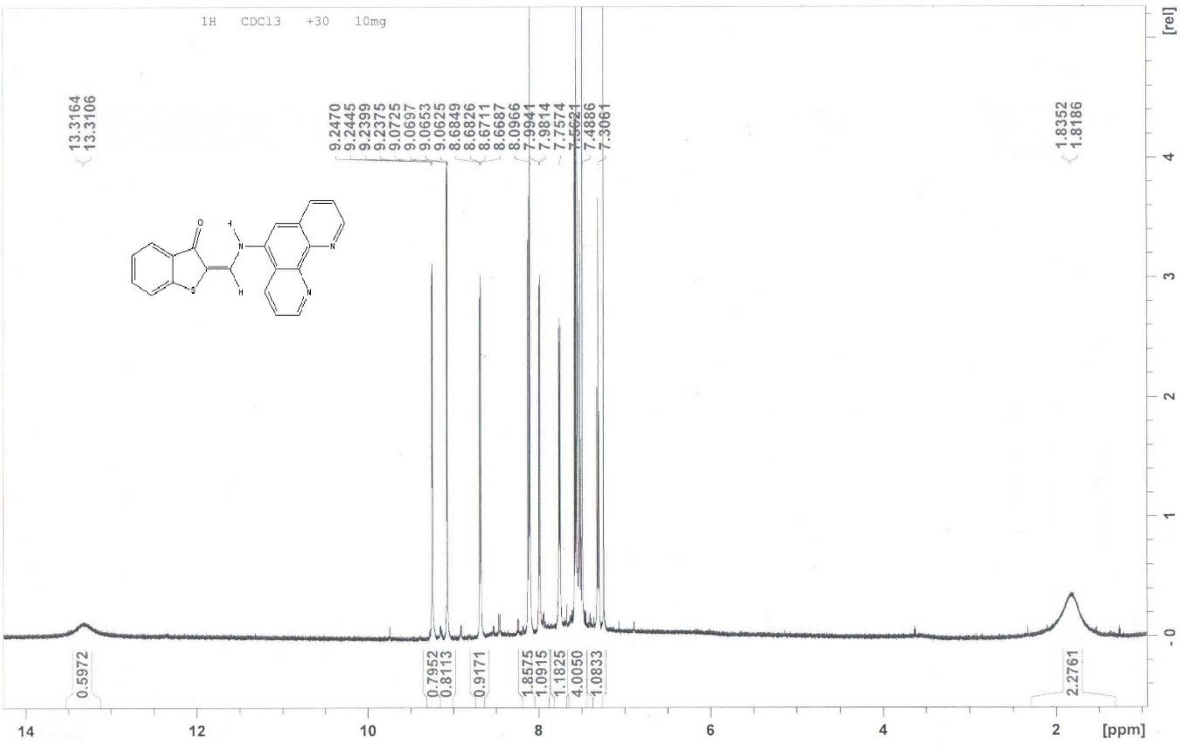

Figure S2: ^{1}H NMR spectrum of compound **2a** in $CDCl_3$.

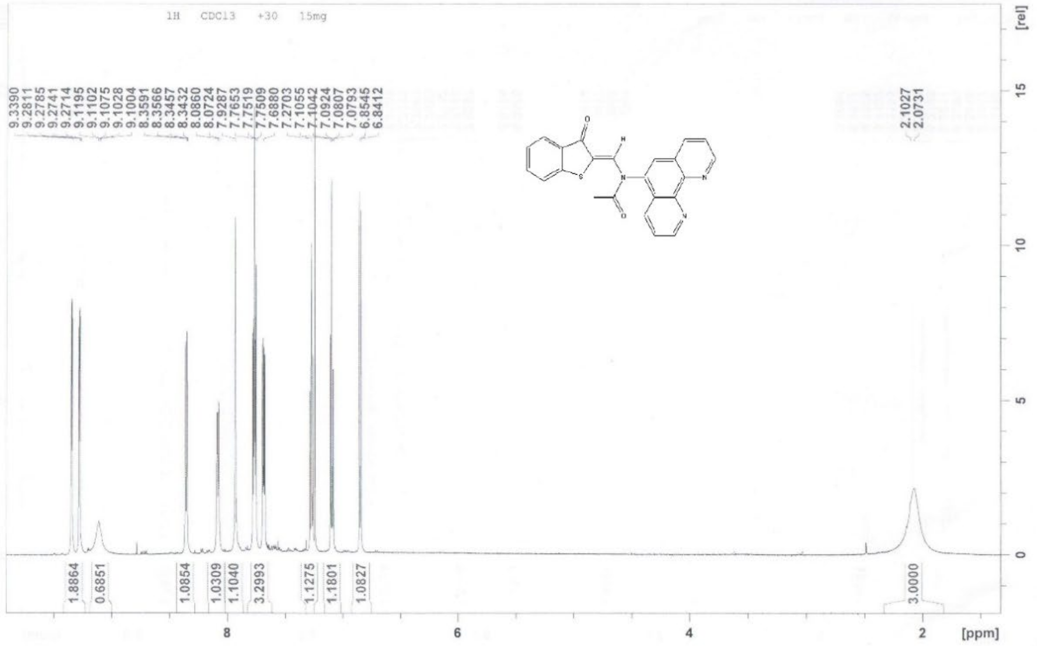

Figure S3: ^{1}H NMR spectrum of compound **2b** in $CDCl_3$.

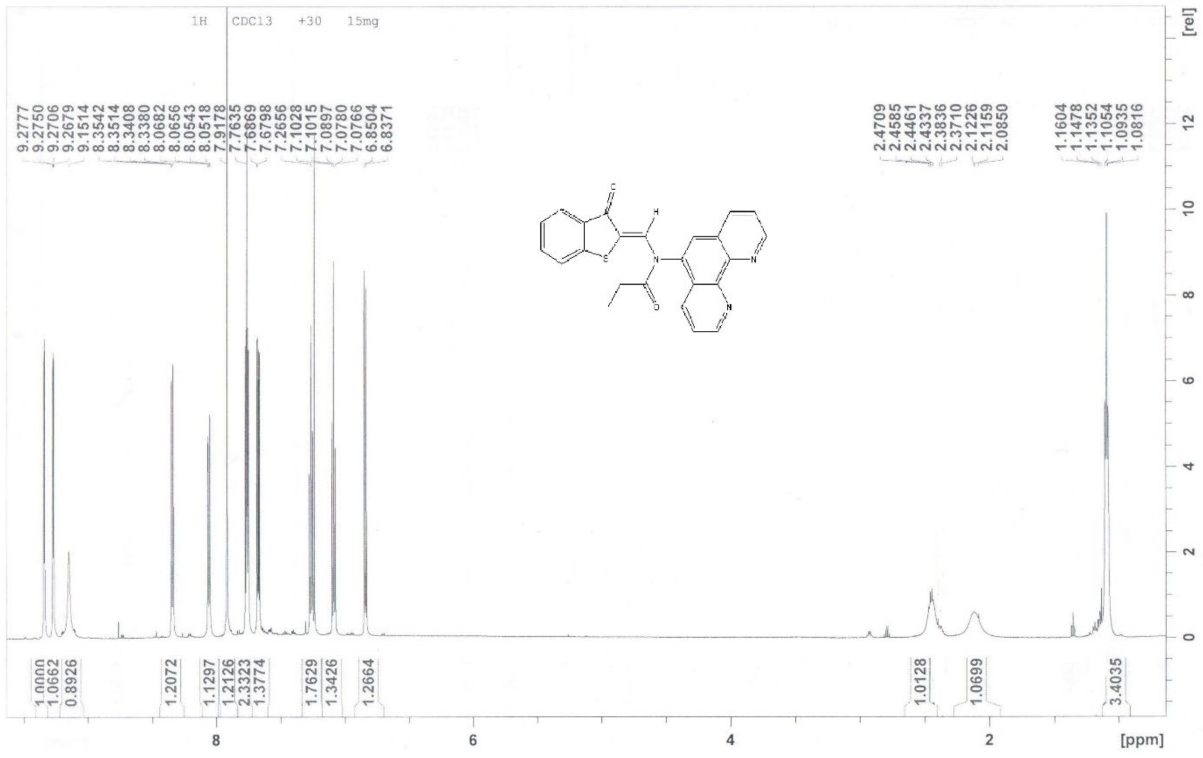

Figure S4: ^{1}H NMR spectrum of compound **2c** in $CDCl_3$.

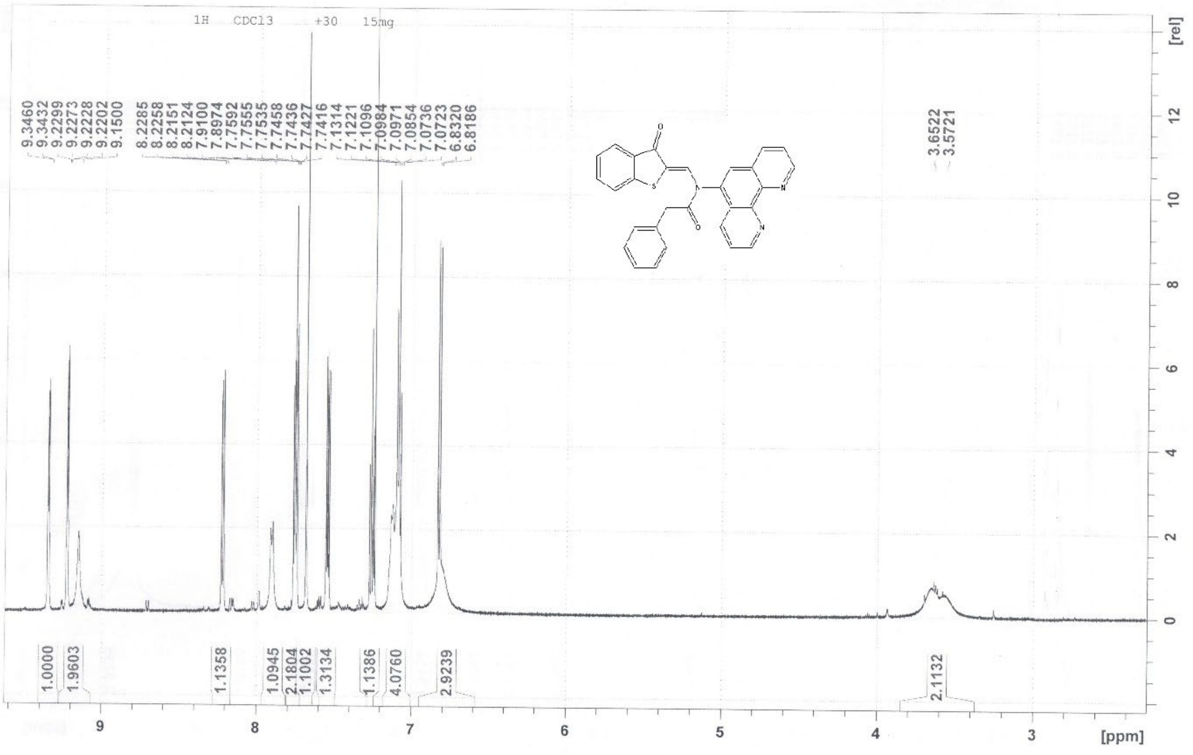

Figure S5: ^{1}H NMR spectrum of compound **3a** in $CDCl_3$.

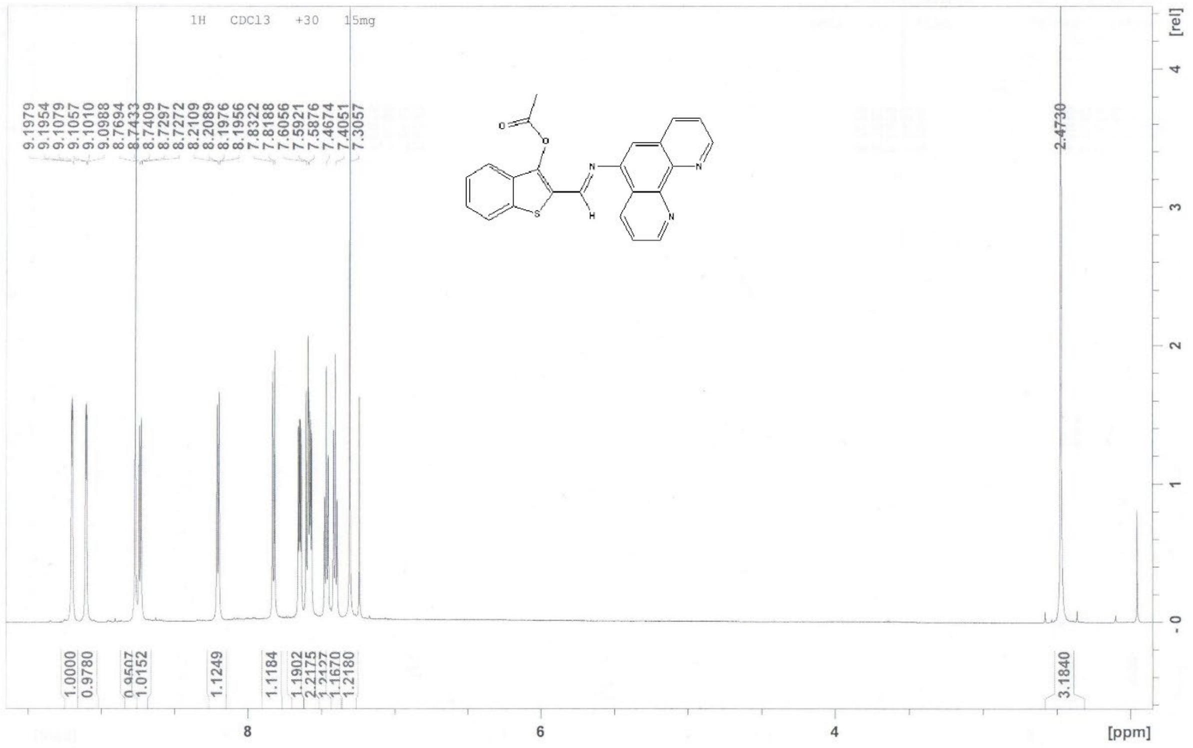

Figure S6: ^{1}H NMR spectrum of compound **3b** in $CDCl_3$.

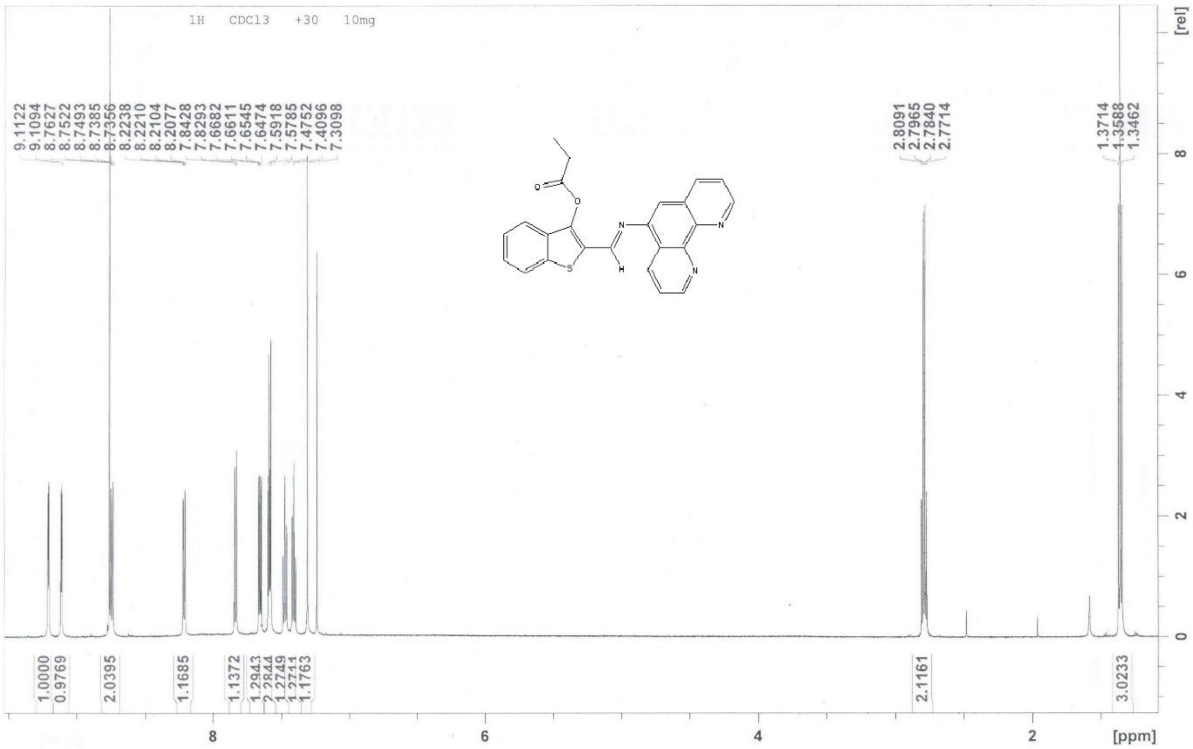

Figure S7: ^{1}H NMR spectrum of compound **3c** in $CDCl_3$.

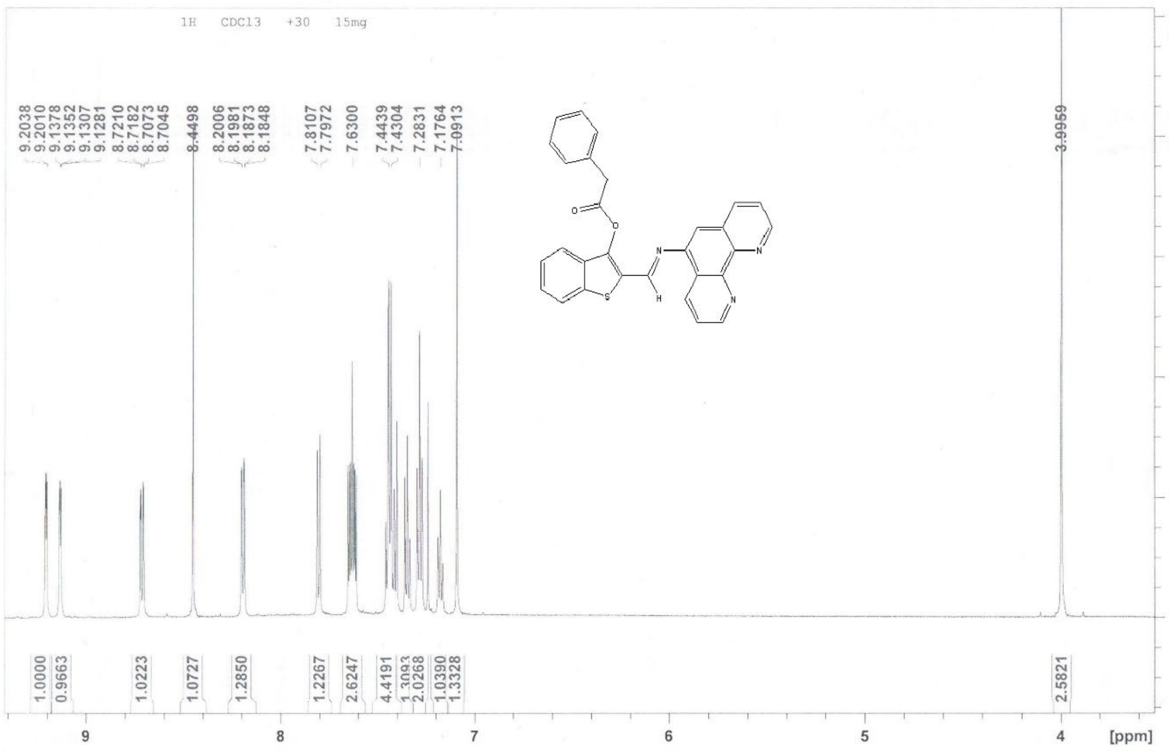

Figure S8: ^{13}C NMR spectrum of compound **1** in $CDCl_3$.

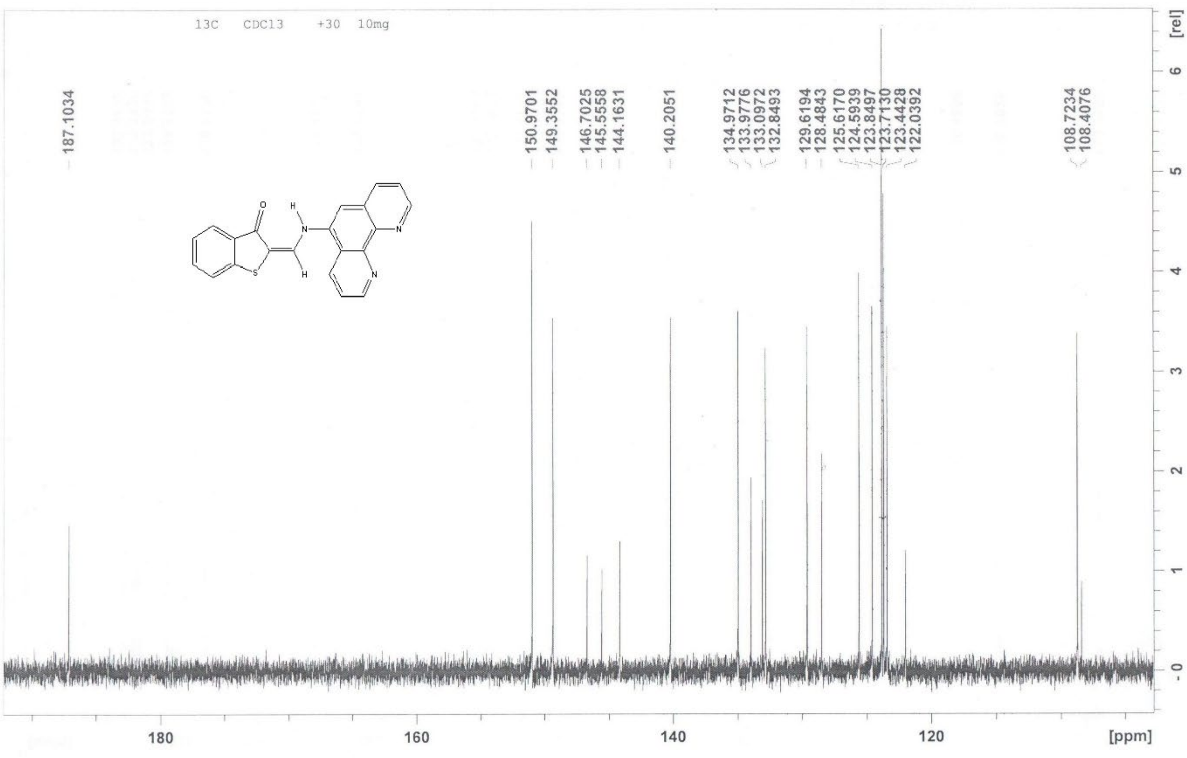

Figure S9: ^{13}C NMR spectrum of compound **2a** in $CDCl_3$.

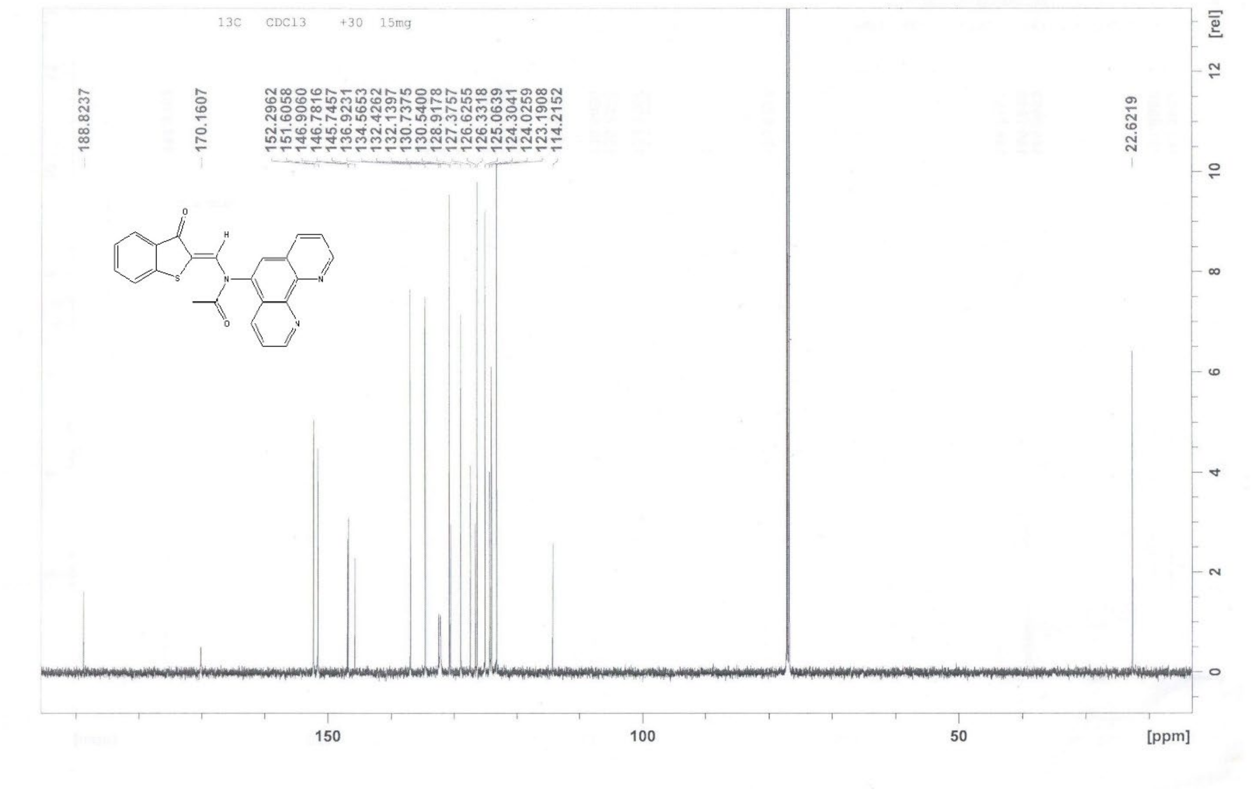

Figure S10: ^{13}C NMR spectrum of compound **2b** in $CDCl_3$.

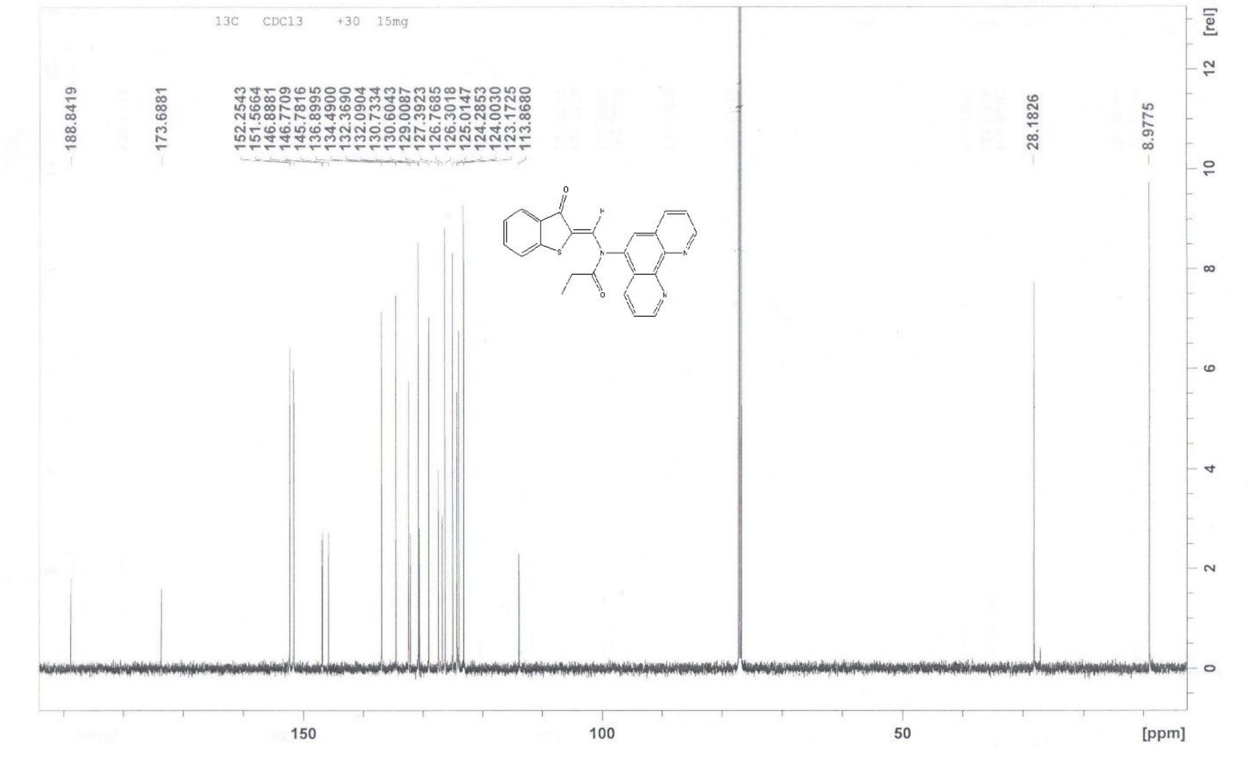

Figure S11: ^{13}C NMR spectrum of compound **2c** in $CDCl_3$.

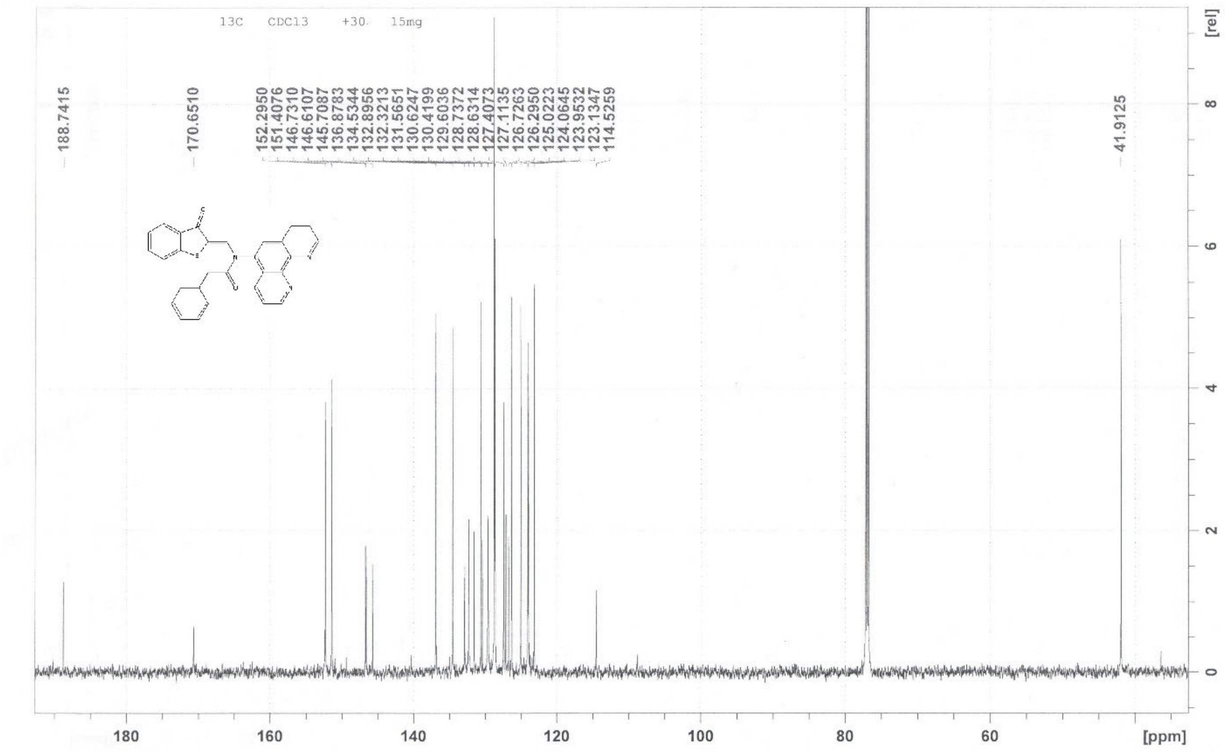

Figure S12: ^{13}C NMR spectrum of compound **3a** in $CDCl_3$.

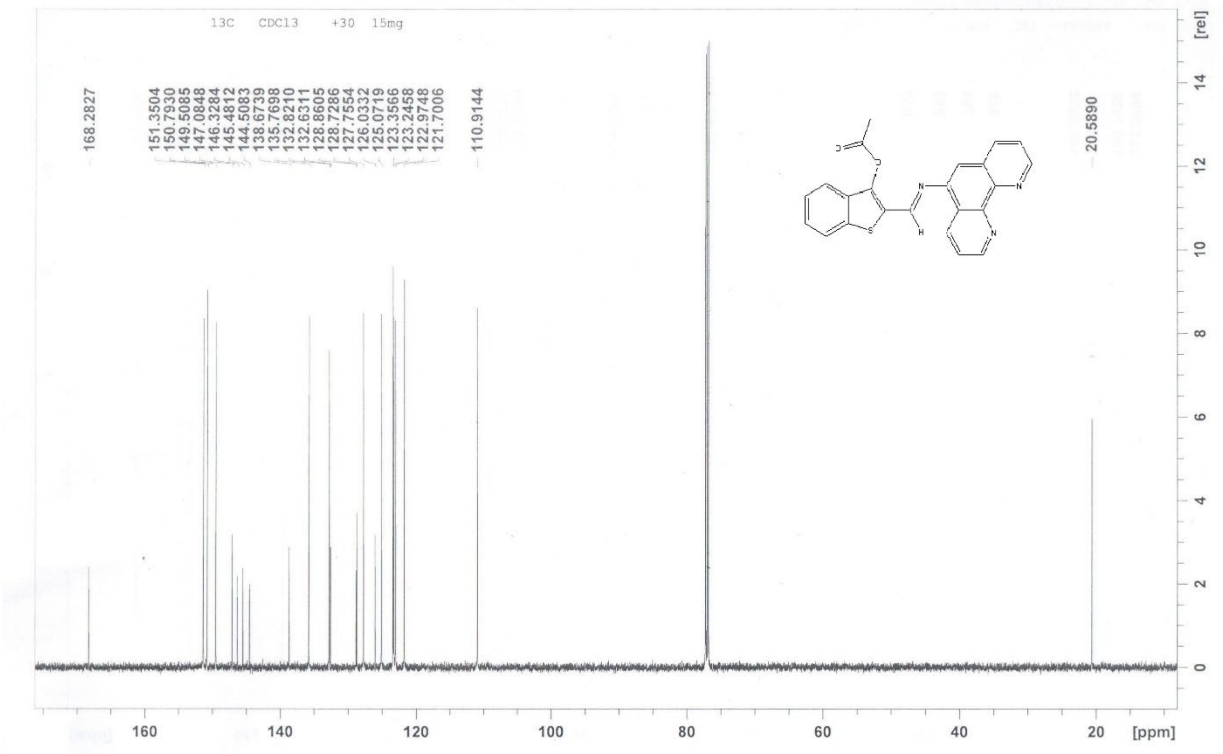

Figure S13: ^{13}C NMR spectrum of compound **3b** in $CDCl_3$.

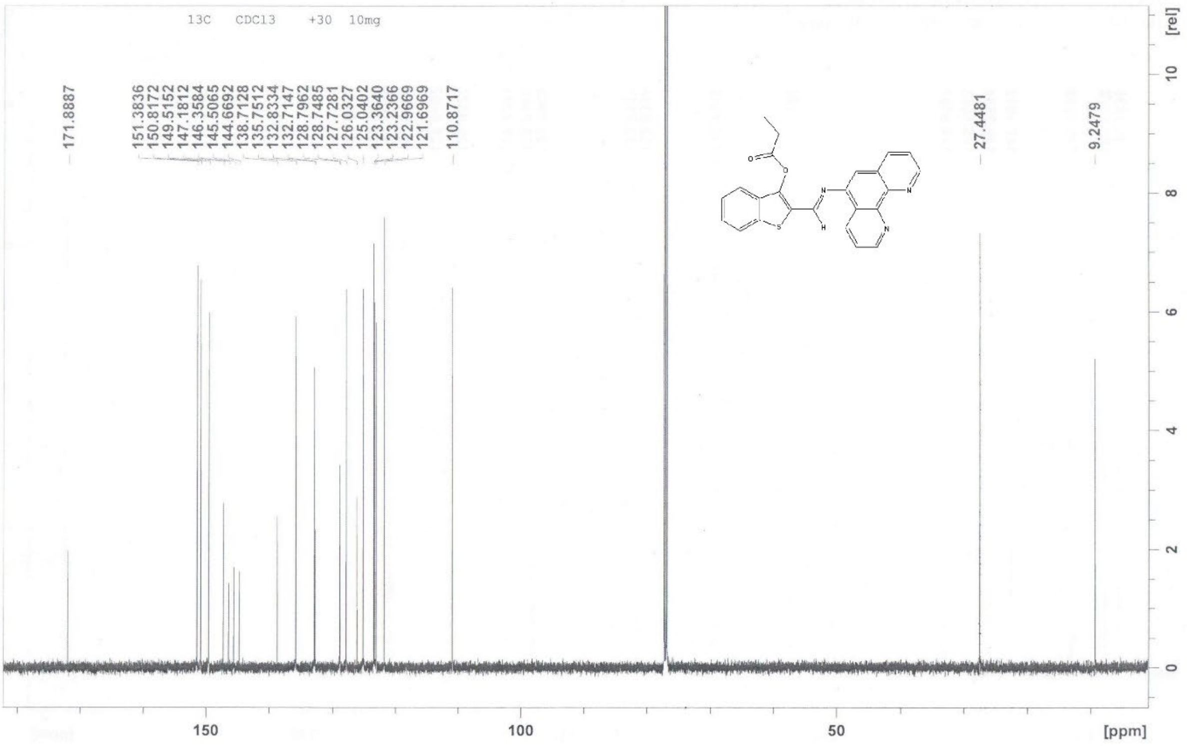

Figure S14: ^{13}C NMR spectrum of compound **3c** in $CDCl_3$.

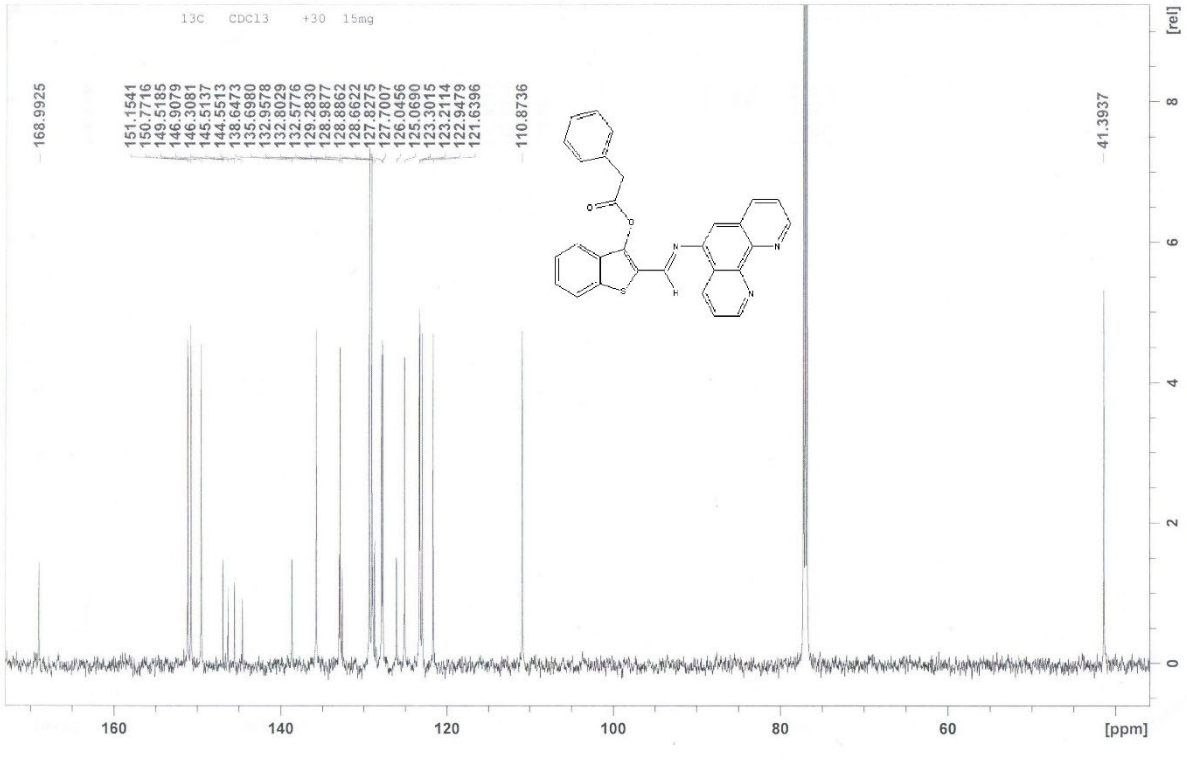

Figure S15: IR spectrum of compound **1**.

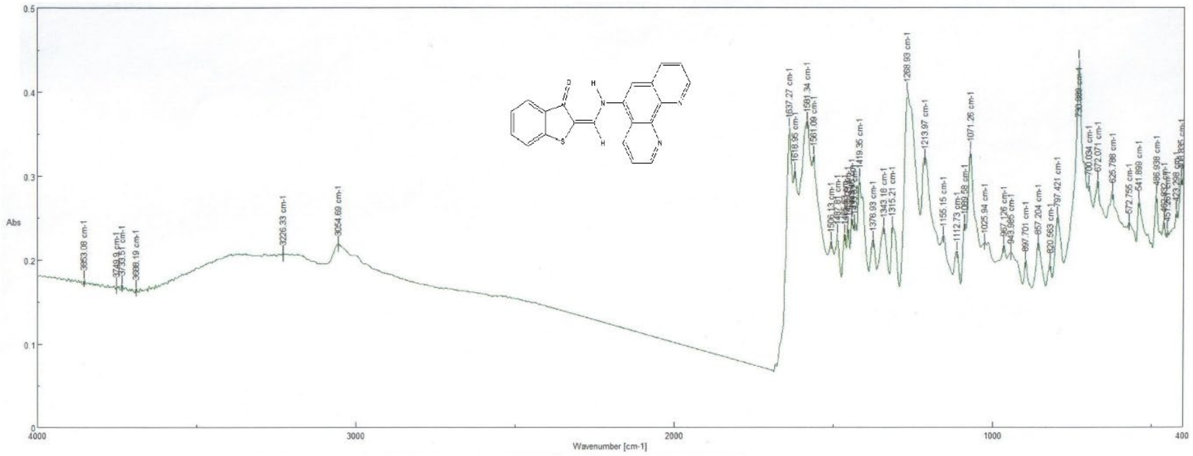

Figure S16: IR spectrum of compound **2a**.

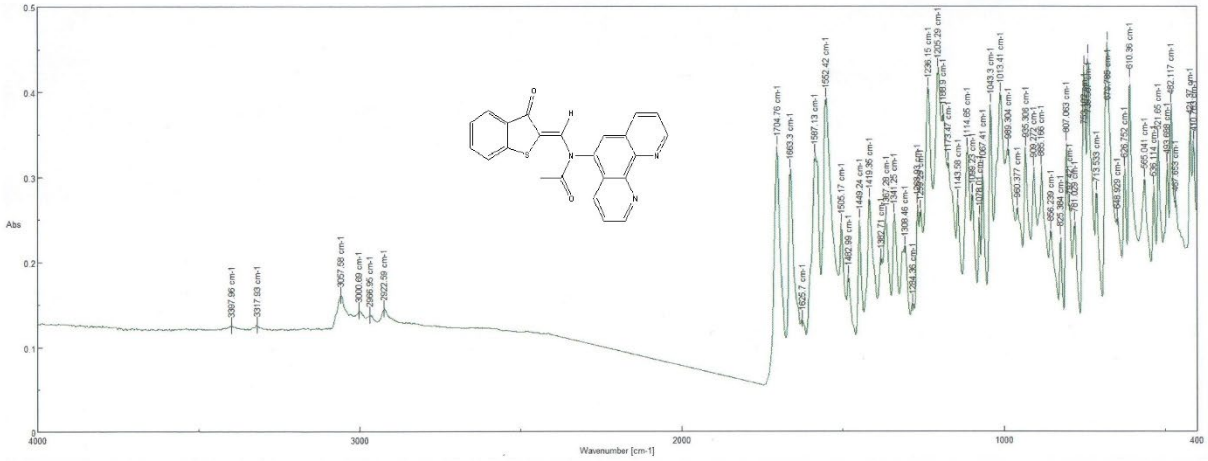

Figure S17: IR spectrum of compound **2b**.

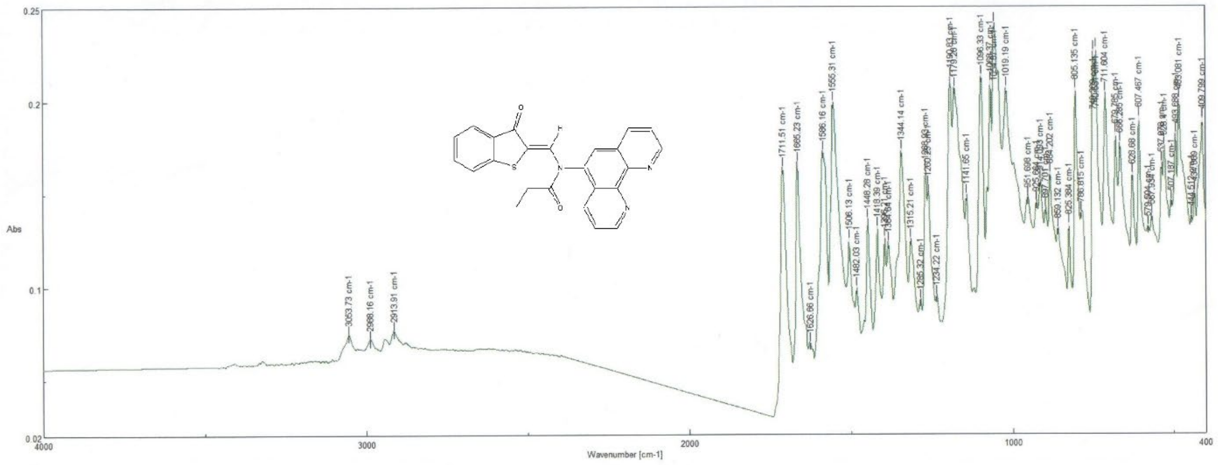

Figure S18: IR spectrum of compound **2c**.

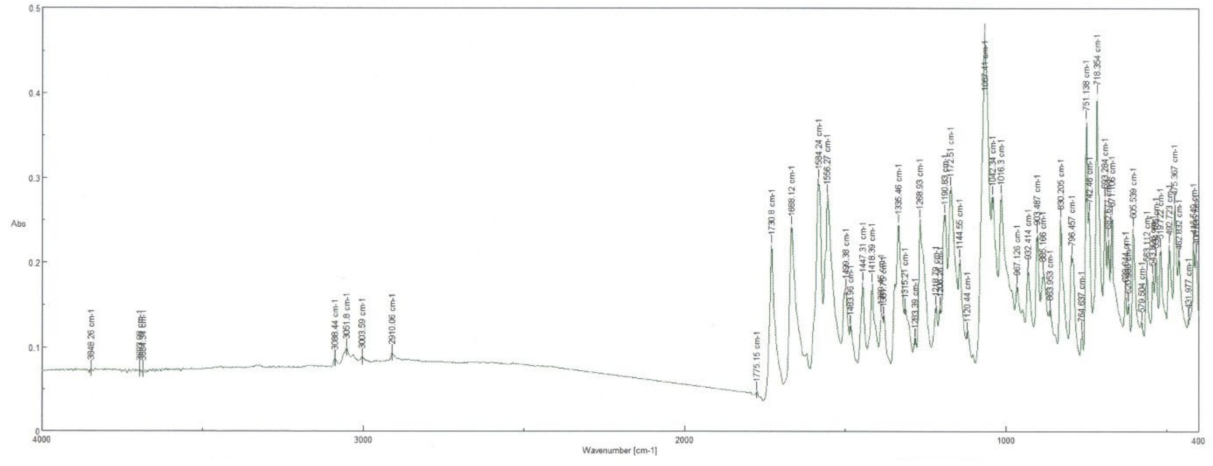

Figure S19: IR spectrum of compound **3a**.

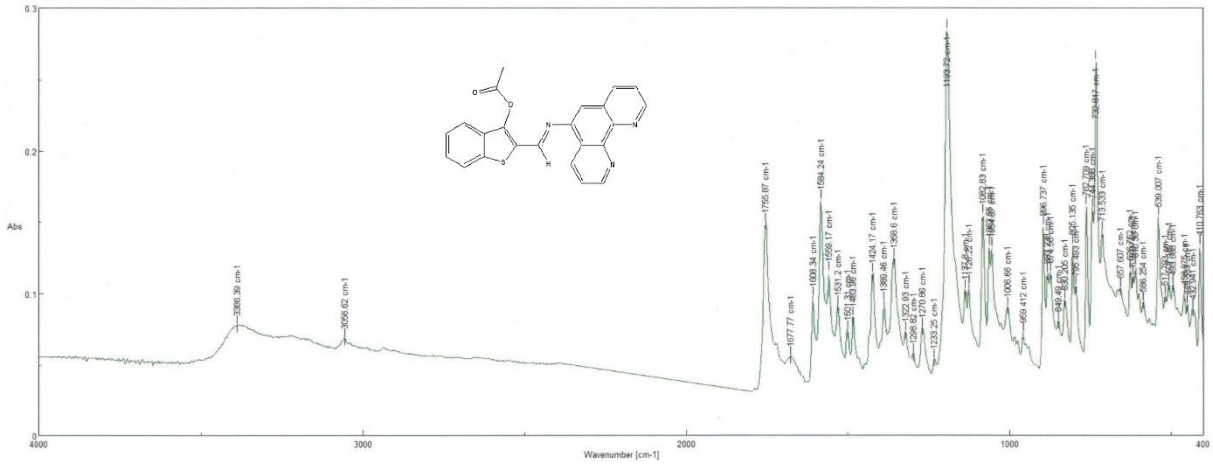

Figure S20: IR spectrum of compound **3b**.

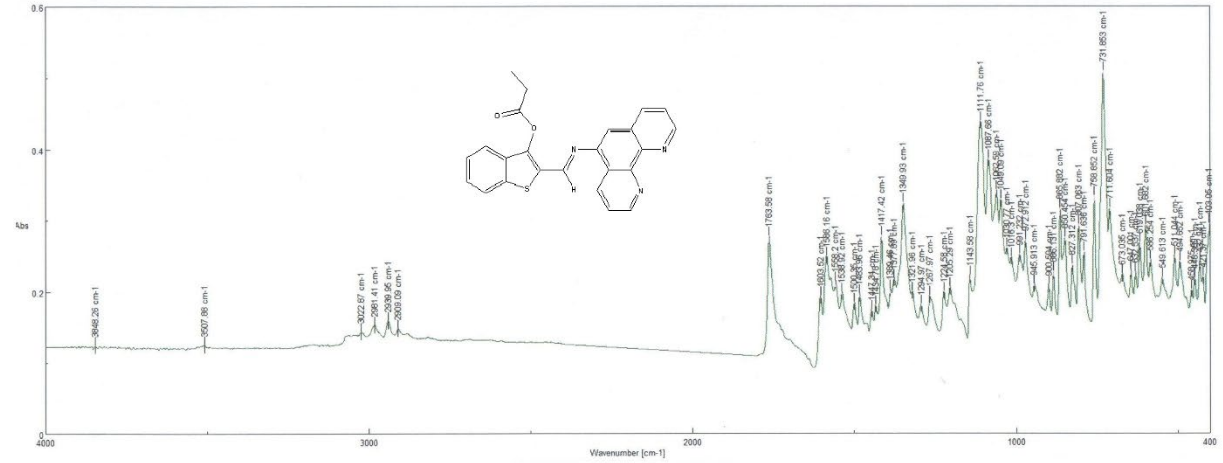

Figure S21: IR spectrum of compound **3c**.

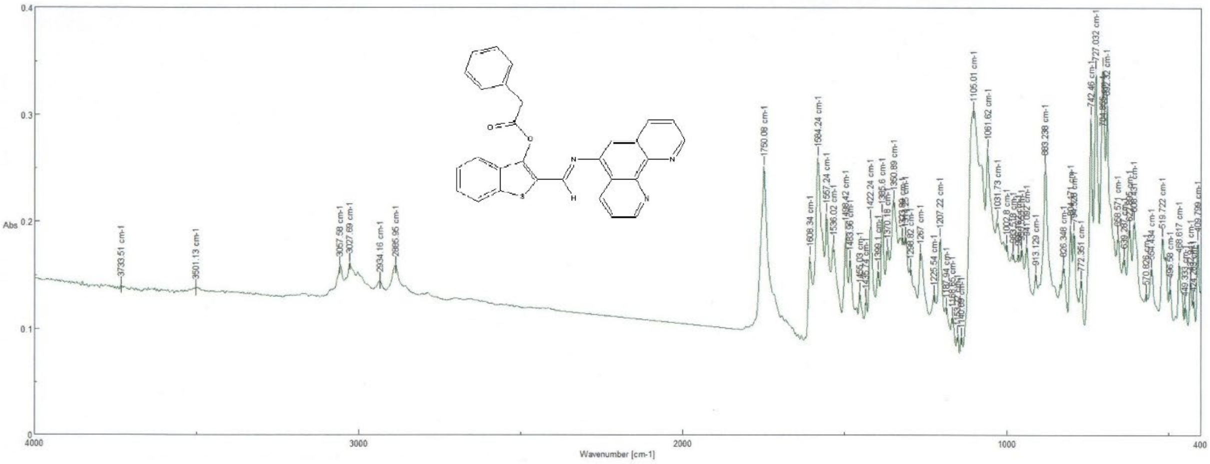

Figure S22: Mass spectrum of compound **1**.

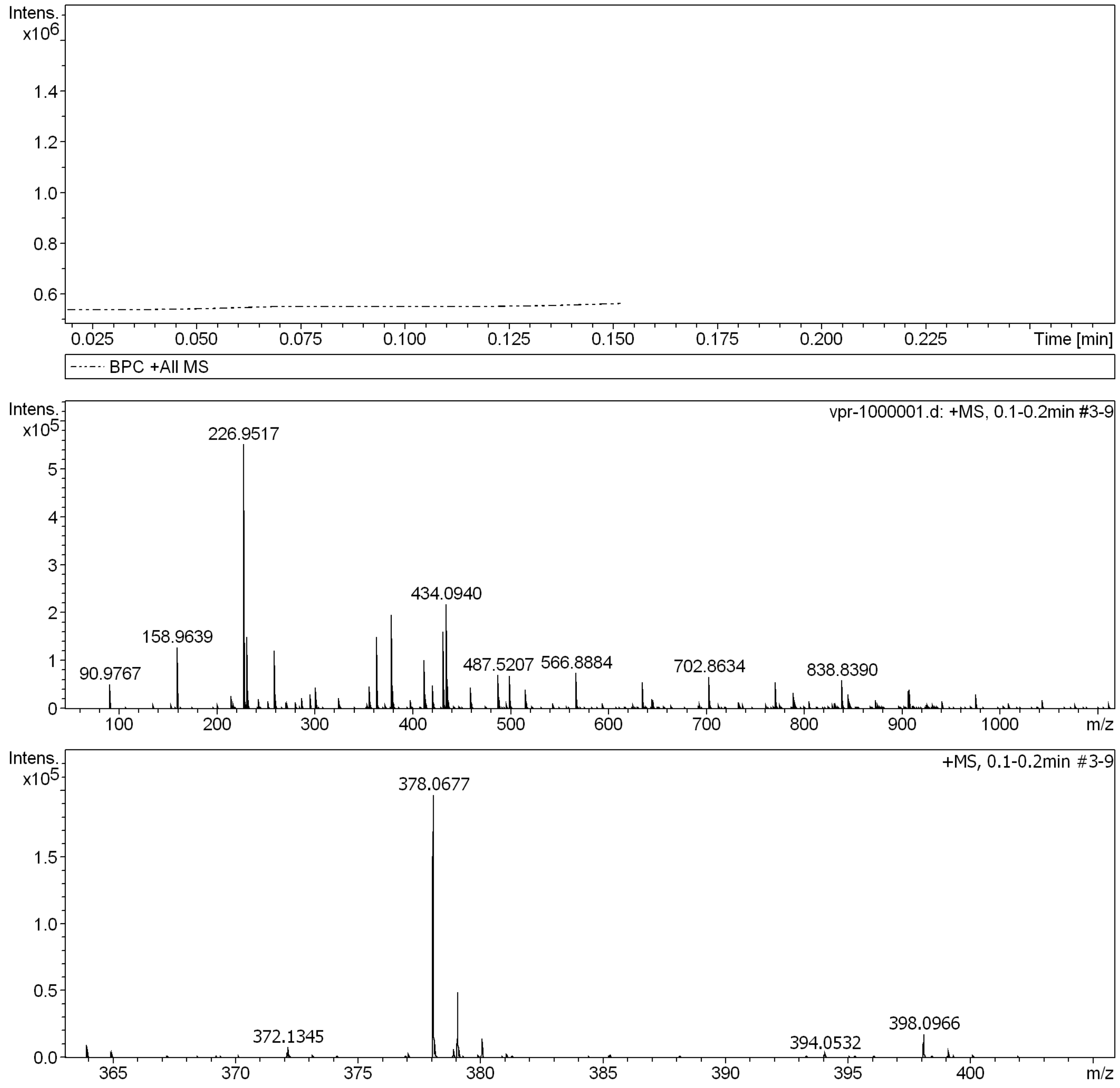

Display Report

Meas. m/z	#	Ion Formula	m/z	err [ppm]	mSigma		Score	rdb	e^- Conf	N-Rule
378.0677	1	C23H12N3OS	378.0696	4.8	10.7	1	100.00	19.5	even	ok
	1	C21H13N3NaOS	378.0672	-1.5	1.6	1	100.00	16.5	even	ok

+MS, 0.1-0.2min #3-9

Figure S23: Mass spectrum of compound **2a**.

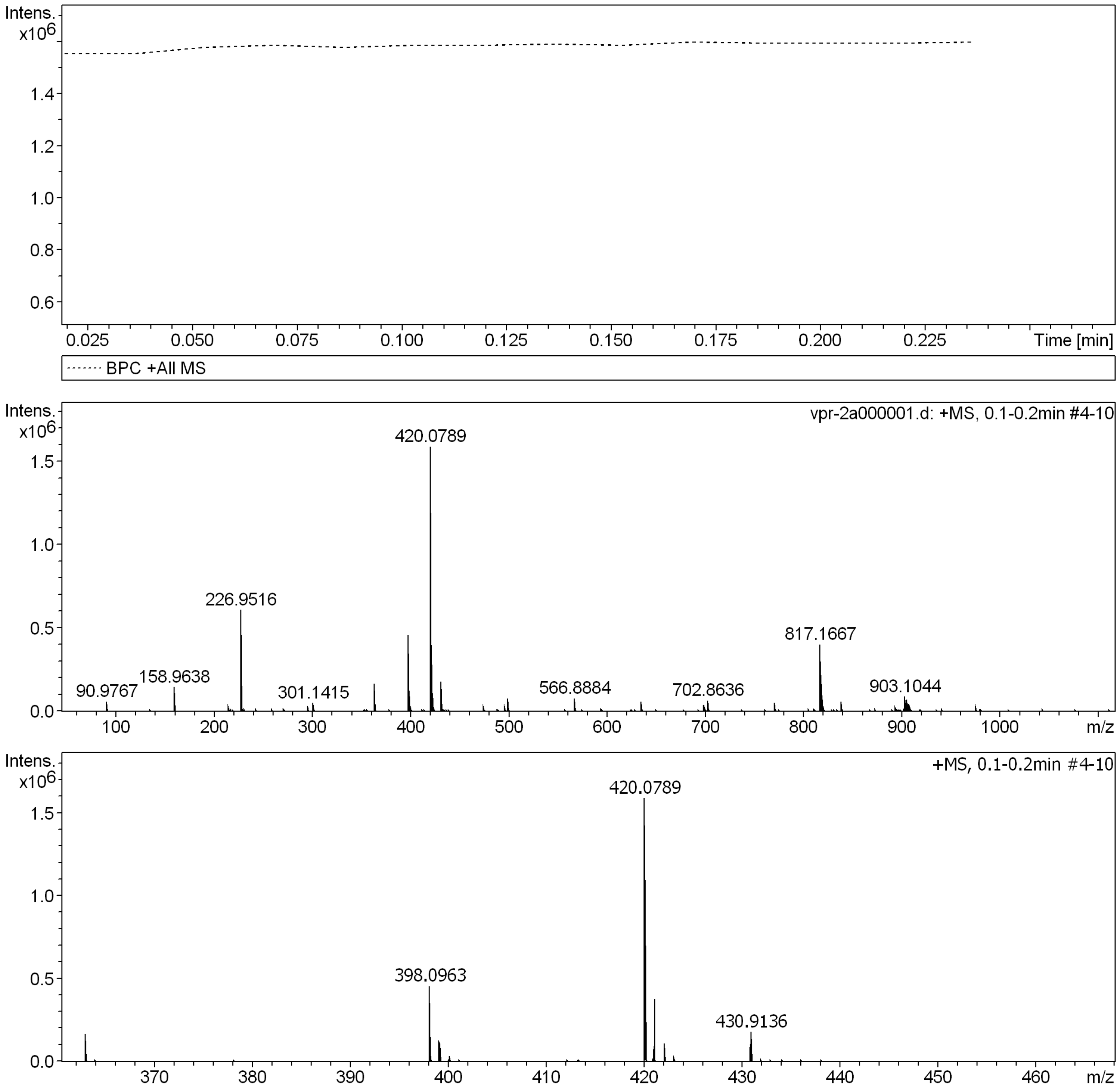

Display Report

Meas. m/z	#	Ion Formula	m/z	err [ppm]	mSigma		Score	rdb	e⁻ Conf	N-Rule
398.0963	1	C23H16N3O2S	398.0958	-1.4	3.4	1	100.00	17.5	even	ok
420.0789	1	C25H14N3O2S	420.0801	3.0	28.6	1	100.00	20.5	even	ok
	1	C23H15N3NaO2S	420.0777	-2.8	17.5	1	100.00	17.5	even	ok

+MS, 0.1-0.2min #4-10

Figure S24: Mass spectrum of compound **2b**.

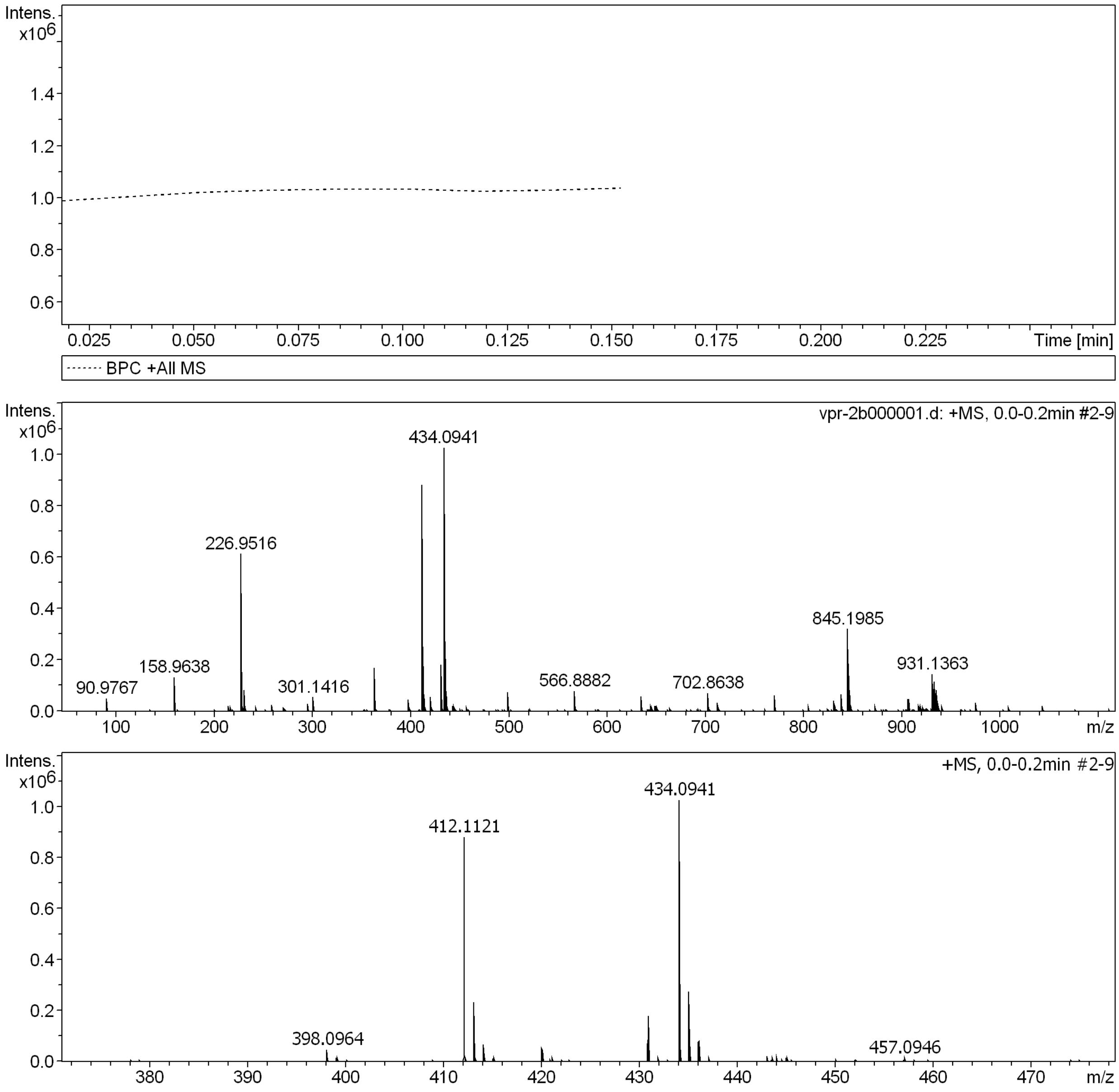

Display Report

Meas. m/z	#	Ion Formula	m/z	err [ppm]	mSigma		Score	rdb	e⁻ Conf	N-Rule
412.1121	1	C24H18N3O2S	412.1114	-1.8	8.6	1	100.00	17.5	even	ok
434.0941	1	C26H16N3O2S	434.0958	3.8	20.2	1	100.00	20.5	even	ok
	1	C24H17N3NaO2S	434.0934	-1.7	9.2	1	100.00	17.5	even	ok

+MS, 0.0-0.2min #2-9

Figure S25: Mass spectrum of compound **2c**.

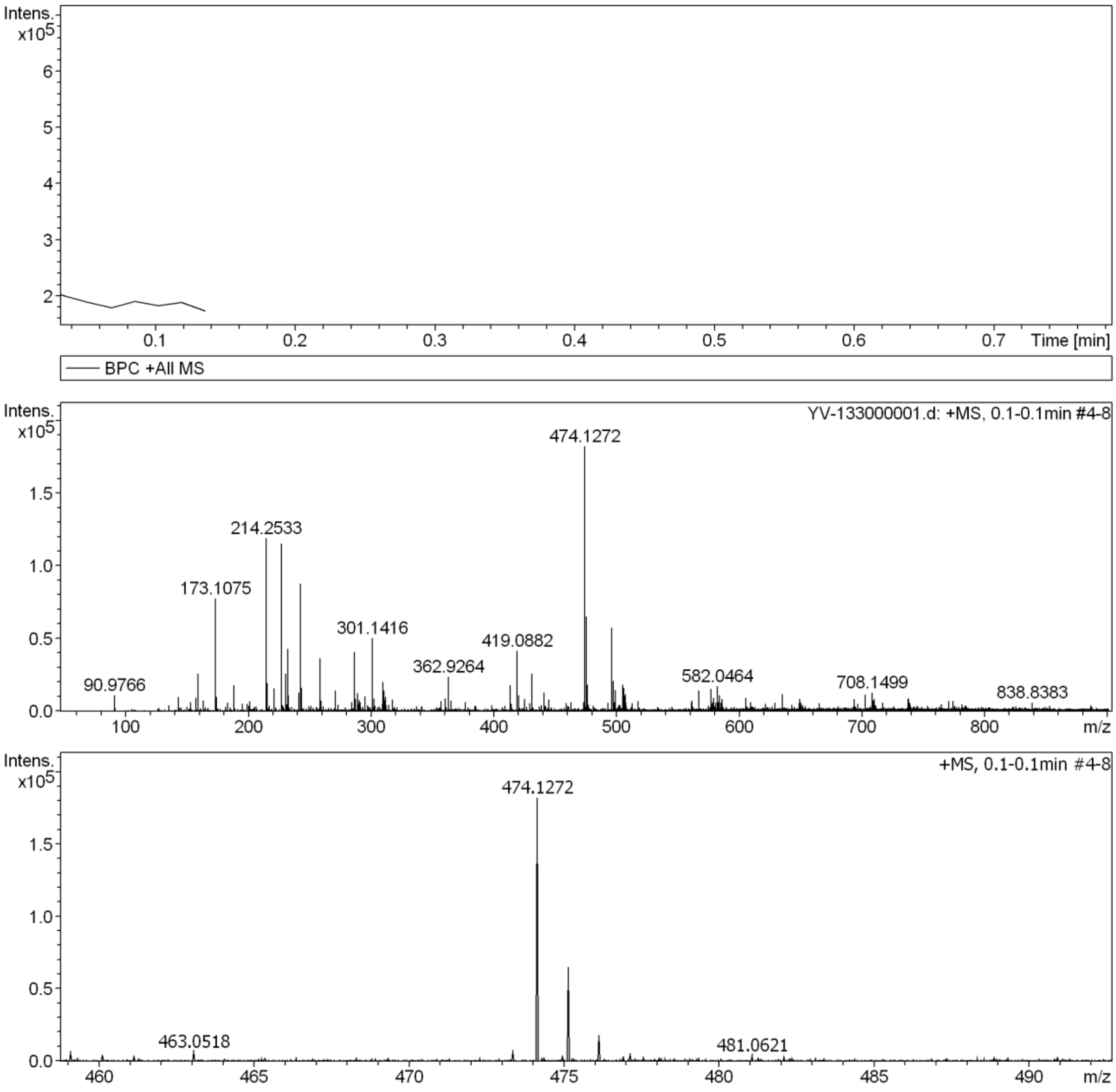

Display Report

Meas. m/z	#	Ion Formula	m/z	err [ppm]	mSigma	#	Score	rdb	e⁻ Conf	N-Rule
474.1272	1	C29H20N3O2S	474.1271	-0.2	11.5	1	100.00	21.5	even	ok
	1	C32H21NNaS	474.1287	3.2	4.4	1	100.00	22.5	even	ok
	1	C29H20N3O2S	474.1271	-0.2	11.5	1	100.00	21.5	even	ok

+MS, 0.1-0.1min #4-8

Figure S26: Mass spectrum of compound **3a**.

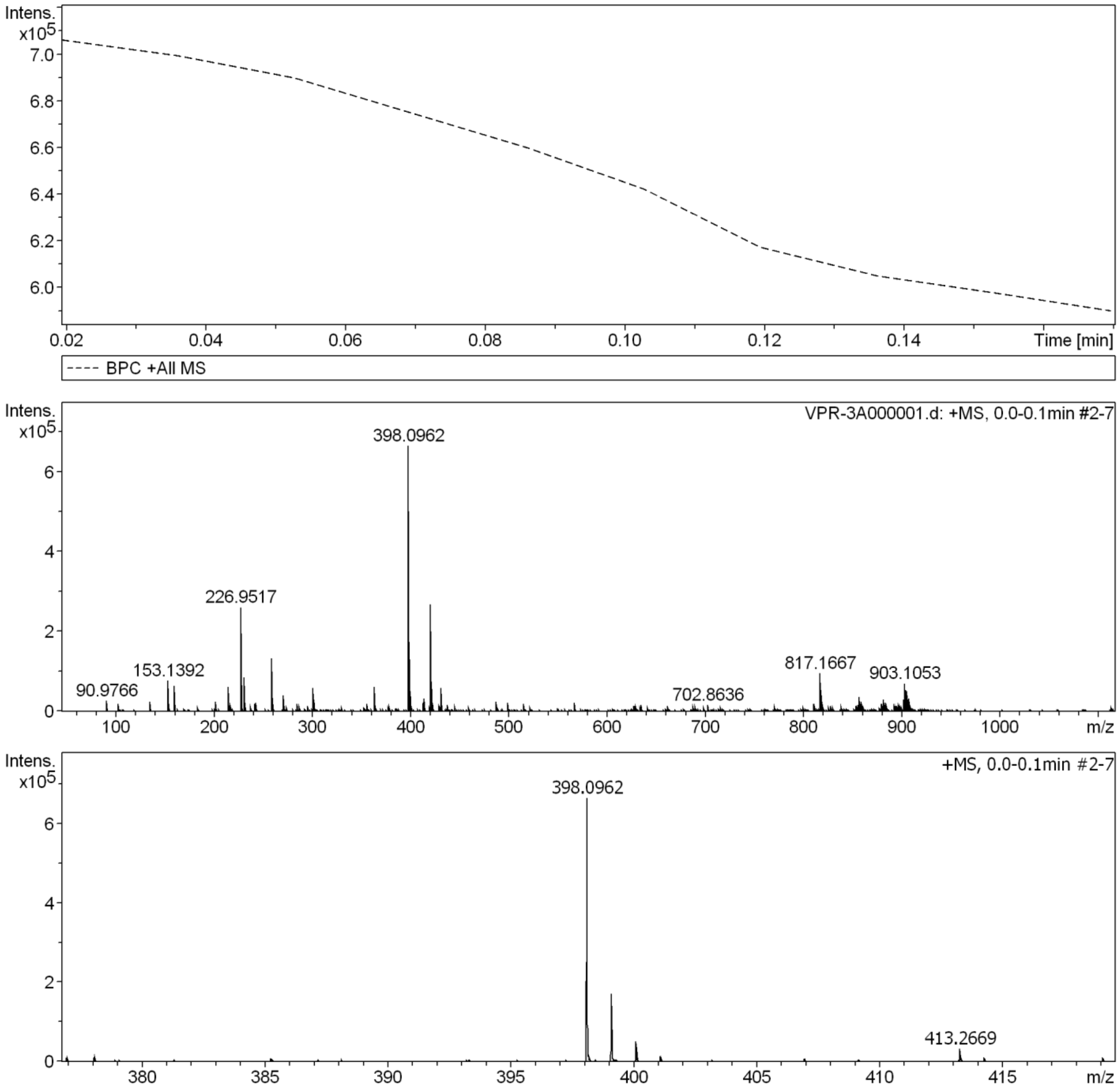

Display Report

Meas. m/z	#	Ion Formula	m/z	err [ppm]	mSigma		Score	rdb	e⁻ Conf	N-Rule
398.0962	1	C23H16N3O2S	398.0958	-1.1	6.3	1	100.00	17.5	even	ok

+MS, 0.0-0.1min #2-7

Figure S27: Mass spectrum of compound **3b**.

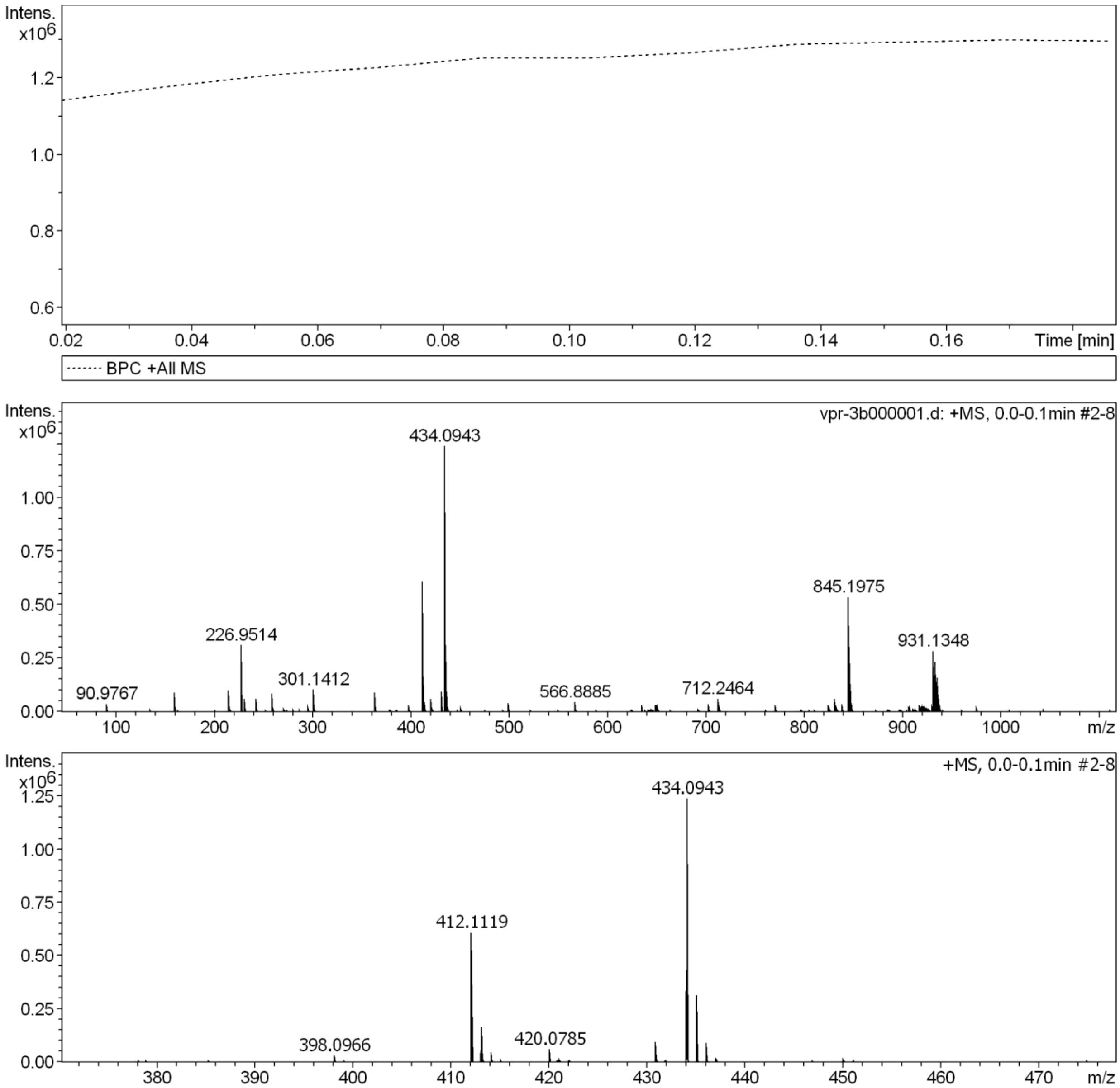

Display Report

Meas. m/z	#	Ion Formula	m/z	err [ppm]	mSigma		Score	rdb	e⁻ Conf	N-Rule
412.1119	1	C24H18N3O2S	412.1114	-1.3	8.0	1	100.00	17.5	even	ok
434.0943	1	C26H16N3O2S	434.0958	3.3	26.7	1	100.00	20.5	even	ok
	1	C24H17N3NaO2S	434.0934	-2.2	15.5	1	100.00	17.5	even	ok

+MS, 0.0-0.1min #2-8

Figure S28: Mass spectrum of compound **3c**.

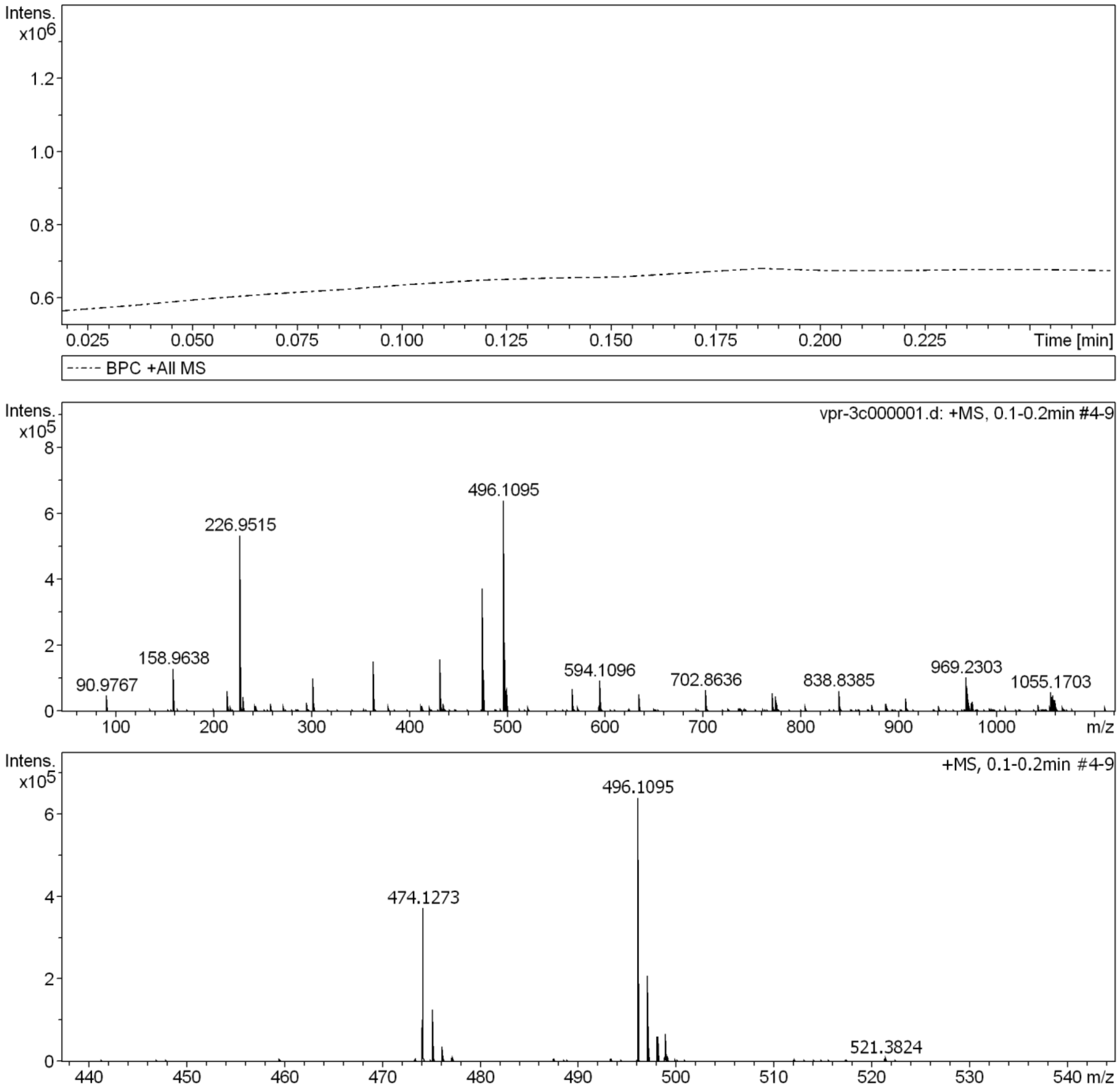

Display Report

Meas. m/z	#	Ion Formula	m/z	err [ppm]	mSigma		Score	rdb	e⁻ Conf	N-Rule
474.1273	1	C29H20N3O2S	474.1271	-0.6	4.1	1	100.00	21.5	even	ok
	1	C24H25N3NaO2S2	474.1280	1.5	28.5	1	100.00	13.5	even	ok
496.1095	1	C31H18N3O2S	496.1114	3.9	16.1	1	100.00	24.5	even	ok
	1	C29H19N3NaO2S	496.1090	-0.9	7.0	1	100.00	21.5	even	ok

+MS, 0.1-0.2min #4-9
