# Supplementary material for: Synthesis of photo- and ionochromic N-acylated 2-(aminomethylene)benzo[b]thiophene-3(2Н)-ones with a terminal phenanthroline group
Source: Beilstein J Org Chem. 2024 Mar 11;20:552–60. doi: 10.3762/bjoc.20.47 (PMC10949002; doi:10.3762/bjoc.20.47)

BEILSTEIN JOURNAL OF ORGANIC CHEMISTRY

Supporting Information

for

Synthesis of photo- and ionochromic N-acylated 2-(aminomethylene)benzo[*b*]thiophene-3(2*H*)-ones with a terminal phenanthroline group

Vladimir P. Rybalkin, Sofiya Yu. Zmeeva, Lidiya L. Popova, Irina V. Dubonosova, Olga Yu. Karlutova, Oleg P. Demidov, Alexander D. Dubonosov and Vladimir A. Bren

Beilstein J. Org. Chem. **2024,** *20,* 552–560. doi:10.3762/bjoc.20.47

X-ray analysis data of 3b

License and Terms: This is a supporting information file under the terms of the Creative Commons Attribution License (https://creativecommons.org/licenses/by/4.0). Please note that the reuse, redistribution and reproduction in particular requires that the author(s) and source are credited and that individual graphics may be subject to special legal provisions.
The license is subject to the *Beilstein Journal of Organic Chemistry* terms and conditions: (https://www.beilstein-journals.org/bjoc/terms)

Table S1: Crystal data and structure refinement for compound **3b**.

CCDC Number	2299603
Empirical formula	$C_{24}H_{17}N_3O_2S$
Formula weight	411.46
Temperature/K	293(2)
Crystal system	triclinic
Space group	*P*-1
a, Å	7.43030(10)
b, Å	9.6398(2)
c, Å	14.3294(3)
α, °	75.731(2)
β, °	82.686(2)
γ, °	78.664(2)
V, $Å^3$	971.93(3)
Z	2
ρ_{calc}, cm^3	1.406
μ, mm^{-1}	1.701
F(000)	428.0
Crystal size, mm	0.361 × 0.281 × 0.1
Radiation	Cu Kα (λ = 1.54184)
2Θ range for data collection, °	9.61 to 152.768
Index ranges	−9 ≤ h ≤ 9, −11 ≤ k ≤ 12, -17 ≤ l ≤ 18
Reflections collected	17777
Independent reflections	4053 [R_{int} = 0.0201, R_σ = 0.0153]
Data/restraints/parameters	4053/0/272
Goodness-of-fit on F2	1.054
Final *R* indices [*I* ≥ 2σ(*I*)]	R_1 = 0.0307, wR_2 = 0.0808
Final *R* indices [all data]	R_1 = 0.0315, wR_2 = 0.0813
Largest diff. peak and hole, e·$Å^{-3}$	0.23/−0.35

Table S2: Bond lengths for compound **3b**.

Atom	Atom	Length/Å	Atom	Atom	Length/Å
S1	C8	1.7409(12)	C15	C14	1.3606(16)
S1	C1	1.7401(11)	C15	C16	1.4292(15)
O1	C2	1.3901(13)	C22	C23	1.4972(16)
O1	C22	1.3820(13)	C9	C1	1.4491(15)
O2	C22	1.2001(14)	C20	C16	1.4147(15)
N3	C14	1.4105(14)	C3	C8	1.4072(17)
N3	C9	1.2827(15)	C3	C4	1.4028(16)
N2	C20	1.3595(14)	C16	C17	1.4086(15)
N2	C19	1.3279(15)	C12	C11	1.3737(17)
N1	C21	1.3607(15)	C17	C18	1.3687(16)
N1	C10	1.3288(15)	C18	C19	1.4046(17)
C13	C21	1.4161(15)	C8	C7	1.3992(16)
C13	C14	1.4466(15)	C10	C11	1.4016(17)
C13	C12	1.4086(15)	C7	C6	1.3833(18)
C2	C3	1.4284(15)	C23	C24	1.5224(17)
C2	C1	1.3605(16)	C4	C5	1.3811(17)
C21	C20	1.4562(15)	C6	C5	1.4003(19)

Table S3: Bond angles for compound **3b**.

Atom	Atom	Atom	Angle/˚	Atom	Atom	Atom	Angle/˚
C1	S1	C8	91.11(6)	C16	C20	C21	118.29(10)
C22	O1	C2	114.92(8)	C8	C3	C2	110.49(10)
C9	N3	C14	117.80(10)	C4	C3	C2	129.47(11)
C19	N2	C20	117.38(10)	C4	C3	C8	120.03(11)
C10	N1	C21	117.48(10)	C20	C16	C15	120.82(10)
C21	C13	C14	120.18(10)	C17	C16	C15	121.33(10)
C12	C13	C21	118.09(10)	C17	C16	C20	117.84(10)
C12	C13	C14	121.71(10)	C11	C12	C13	119.50(10)
O1	C2	C3	123.91(10)	C18	C17	C16	119.58(10)
C1	C2	O1	121.64(10)	C17	C18	C19	118.33(10)
C1	C2	C3	114.44(10)	C3	C8	S1	112.08(8)

N1	C21	C13	122.20(10)	C7	C8	S1	126.93(10)
N1	C21	C20	118.31(10)	C7	C8	C3	120.99(11)
C13	C21	C20	119.47(10)	C2	C1	S1	111.83(8)
C14	C15	C16	121.22(10)	C2	C1	C9	126.95(10)
N3	C14	C13	117.43(10)	C9	C1	S1	121.21(9)
C15	C14	N3	122.44(10)	N2	C19	C18	124.33(10)
C15	C14	C13	119.95(10)	N1	C10	C11	124.56(11)
O1	C22	C23	111.09(9)	C6	C7	C8	117.89(12)
O2	C22	O1	121.99(10)	C22	C23	C24	110.62(10)
O2	C22	C23	126.91(10)	C12	C11	C10	118.15(11)
N3	C9	C1	120.05(10)	C5	C4	C3	118.80(12)
N2	C20	C21	119.19(10)	C7	C6	C5	121.63(11)
N2	C20	C16	122.51(10)	C4	C5	C6	120.65(11)

Table S4: Torsion angles for compound **3b**.

A	B	C	D	Angle/˚	A	B	C	D	Angle/˚
S1	C8	C7	C6	179.44(9)	C14	C15	C16	C17	-179.41(10)
O1	C2	C3	C8	-179.17(10)	C22	O1	C2	C3	-75.26(13)
O1	C2	C3	C4	1.65(19)	C22	O1	C2	C1	105.89(12)
O1	C2	C1	S1	177.69(8)	C9	N3	C14	C13	-139.08(10)
O1	C2	C1	C9	-1.18(17)	C9	N3	C14	C15	45.83(15)
O1	C22	C23	C24	170.73(9)	C20	N2	C19	C18	-0.60(17)
O2	C22	C23	C24	-10.02(17)	C20	C16	C17	C18	-0.96(16)
N3	C9	C1	S1	10.30(15)	C3	C2	C1	S1	-1.26(13)
N3	C9	C1	C2	-170.92(11)	C3	C2	C1	C9	179.87(10)
N2	C20	C16	C15	-177.59(10)	C3	C8	C7	C6	-1.03(17)
N2	C20	C16	C17	1.57(16)	C3	C4	C5	C6	-0.36(18)
N1	C21	C20	N2	-4.53(15)	C16	C15	C14	N3	173.51(10)
N1	C21	C20	C16	175.49(10)	C16	C15	C14	C13	-1.46(16)
N1	C10	C11	C12	1.50(19)	C16	C17	C18	C19	-0.31(17)
C13	C21	C20	N2	177.20(10)	C12	C13	C21	N1	1.30(16)
C13	C21	C20	C16	-2.78(15)	C12	C13	C21	C20	179.51(10)
C13	C12	C11	C10	-0.33(17)	C12	C13	C14	N3	7.48(15)
C2	O1	C22	O2	6.67(14)	C12	C13	C14	C15	-177.31(10)

C2	O1	C22	C23	-174.04(9)	C17	C18	C19	N2	1.17(18)
C2	C3	C8	S1	1.64(12)	C8	S1	C1	C2	1.84(9)
C2	C3	C8	C7	-177.96(10)	C8	S1	C1	C9	-179.21(9)
C2	C3	C4	C5	178.52(11)	C8	C3	C4	C5	-0.59(17)
C21	N1	C10	C11	-1.20(18)	C8	C7	C6	C5	0.07(18)
C21	C13	C14	N3	-174.20(9)	C1	S1	C8	C3	-1.99(9)
C21	C13	C14	C15	1.01(16)	C1	S1	C8	C7	177.58(11)
C21	C13	C12	C11	-0.97(16)	C1	C2	C3	C8	-0.24(14)
C21	C20	C16	C15	2.39(15)	C1	C2	C3	C4	-179.43(11)
C21	C20	C16	C17	-178.45(10)	C19	N2	C20	C21	179.22(10)
C15	C16	C17	C18	178.20(10)	C19	N2	C20	C16	-0.80(16)
C14	N3	C9	C1	-172.99(9)	C10	N1	C21	C13	-0.24(16)
C14	C13	C21	N1	-177.08(10)	C10	N1	C21	C20	-178.47(10)
C14	C13	C21	C20	1.13(15)	C7	C6	C5	C4	0.64(19)
C14	C13	C12	C11	177.39(10)	C4	C3	C8	S1	-179.09(9)
C14	C15	C16	C20	-0.28(16)	C4	C3	C8	C7	1.31(17)

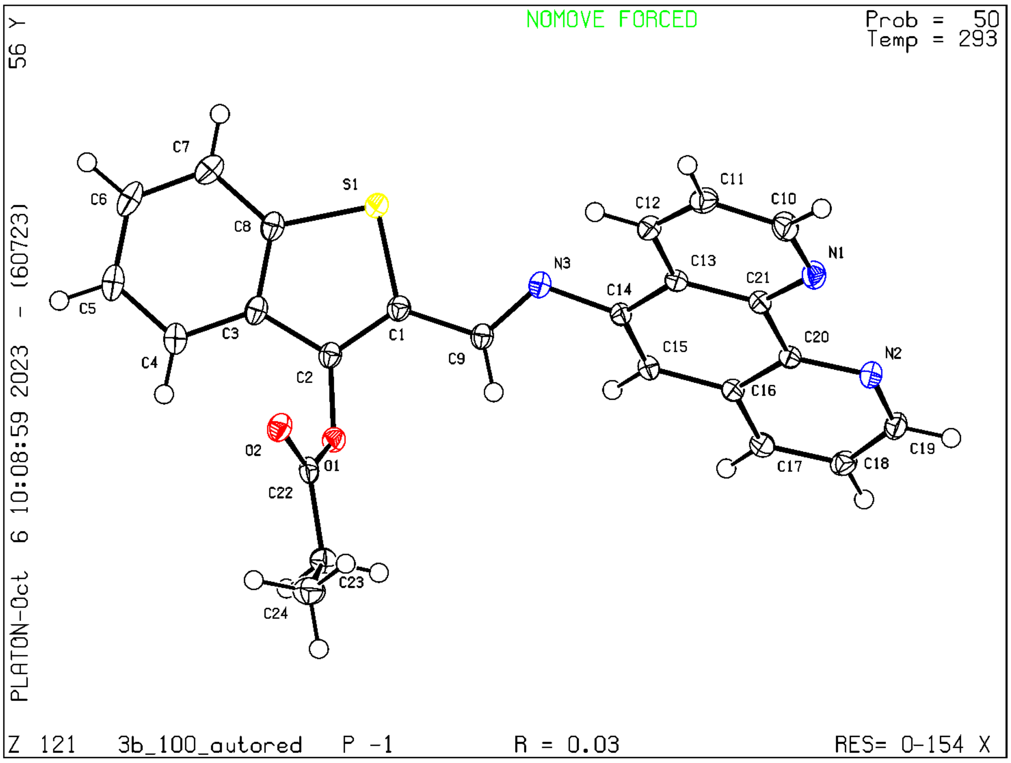

Supplement: File 3 — X-ray analysis data of 3b. [file Beilstein_J_Org_Chem-20-552-s003.pdf]
